# Systematic Review on Polyphenol Intake and Health Outcomes: Is there Sufficient Evidence to Define a Health-Promoting Polyphenol-Rich Dietary Pattern?

**DOI:** 10.3390/nu11061355

**Published:** 2019-06-16

**Authors:** Cristian Del Bo’, Stefano Bernardi, Mirko Marino, Marisa Porrini, Massimiliano Tucci, Simone Guglielmetti, Antonio Cherubini, Barbara Carrieri, Benjamin Kirkup, Paul Kroon, Raul Zamora-Ros, Nicole Hidalgo Liberona, Cristina Andres-Lacueva, Patrizia Riso

**Affiliations:** 1 Department of Food, Environmental and Nutritional Sciences (DeFENS), Università degli Studi di Milano, 20133 Milan, Italy; cristian.delbo@unimi.it (C.D.B.); stefano.bernardi@unimi.it (S.B.); mirko.marino@unimi.it (M.M.); marisa.porrini@unimi.it (M.P.); massimiliano.tucci.mt@gmail.com (M.T.); simone.guglielmetti@unimi.it (S.G.); 2 Geriatria, Accettazione Geriatrica e Centro di ricerca per l’invecchiamento, IRCCS INRCA, 60127 Ancona, Italy; a.cherubini@inrca.it (A.C.); carrieribarbara@gmail.com (B.C.); 3 Department of Life and Environmental Sciences, Polytechnic University of Marche, Via Brecce Bianche, 60131 Ancona, Italy; 4 Quadram Institute Bioscience, Norwich Research Park, Norwich NR4 7UG, UK; benjamin.kirkup@quadram.ac.uk (B.K.); paul.kroon@quadram.ac.uk (P.K.); 5 Unit of Nutrition and Cancer, Cancer Epidemiology Research Programme, Catalan Institute of Oncology (ICO), Bellvitge Biomedical Research Institute (IDIBELL), 08908 L’Hospitalet de Llobregat, Spain; rzamora@idibell.cat; 6 Biomarkers and Nutrimetabolomics Laboratory, Department of Nutrition, Food Sciences and Gastronomy, XaRTA, INSA, Faculty of Pharmacy and Food Sciences, University of Barcelona, 08028 Barcelona, Spain; n.hidalgoliberona@ub.edu (N.H.L.); candres@ub.edu (C.A.-L.); 7 CIBER Fragilidad y Envejecimiento Saludable (CIBERfes), Instituto de Salud Carlos III, 28029 Madrid, Spain

**Keywords:** polyphenol intake, polyphenol databases, dietary pattern, disease risk, cardiovascular and all-cause mortality

## Abstract

Growing evidence support association between polyphenol intake and reduced risk for chronic diseases, even if there is a broad debate about the effective amount of polyphenols able to exert such protective effect. The present systematic review provides an overview of the last 10-year literature on the evaluation of polyphenol intake and its association with specific disease markers and/or endpoints. An estimation of the mean total polyphenol intake has been performed despite the large heterogeneity of data reviewed. In addition, the contribution of dietary sources was considered, suggesting tea, coffee, red wine, fruit and vegetables as the main products providing polyphenols. Total flavonoids and specific subclasses, but not total polyphenols, have been apparently associated with a low risk of diabetes, cardiovascular events and all-cause mortality. However, large variability in terms of methods for the evaluation and quantification of polyphenol intake, markers and endpoints considered, makes it still difficult to establish an evidence-based reference intake for the whole class and subclass of compounds. Nevertheless, the critical mass of data available seem to strongly suggest the protective effect of a polyphenol-rich dietary pattern even if further well targeted and methodologically sound research should be encouraged in order to define specific recommendations.

## 1. Introduction

The possibility to develop dietary guidelines for the intake of food bioactives with health promoting effects can be of utmost importance to try to evolve the concept of adequate nutrition to that of optimal nutrition. Clearly, this implies at least 2 levels of knowledge: (1) the availability of reliable data of food composition and food intake to estimate exposure to food bioactives and (2) the capacity to assess the amount needed to exert the protective activity.

Polyphenols have been suggested to exert a plethora of biological activities including antioxidant, anti-inflammatory, anti-microbial, anti-proliferative, pro-apoptotic activity and hormonal regulation capacity [1]. There is also increasing evidence that long-term intake can have favorable effects on the incidence of several cancers and other chronic diseases, including cardiovascular disease (CVD), type II diabetes, and neurodegenerative diseases [2]. More recently research has been focused on the impact of polyphenols on healthy aging and/or age-related diseases [3].

**Figure 1:**
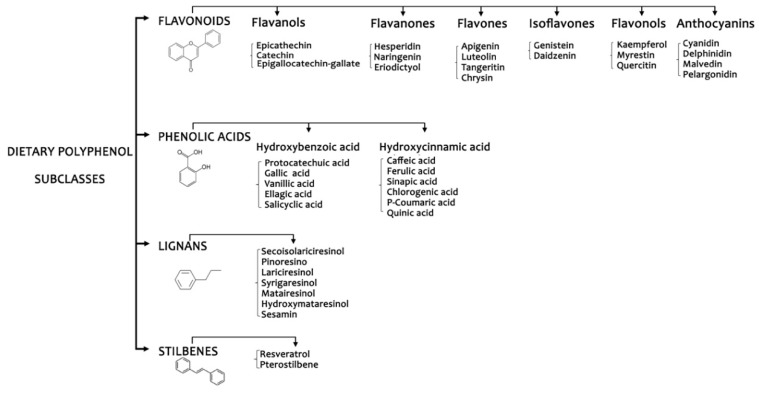
Polyphenol subclasses.

The emerging evidence, obtained through both animal models and human studies, on the direct and indirect role of polyphenols in the modulation of metabolic and functional features of the host, has enhanced the interest for an estimation of polyphenol intake in the general population or in at risk target groups. In addition, the assessment of specificity in the protective properties of the single polyphenol classes/compounds (Figure 1) has been increased in the last years favored by the improvement of dedicated food databases (i.e., Phenol-Explorer, USDA database) reporting more accurate and detailed polyphenols composition and considering factors affecting the intake such as the “retention factors” (i.e., the loss or gain of a compound during food processing). Despite the transformation of food intake data into polyphenol intake remains still a critical, even if improved, step of the process, the accuracy of self-reported methods to evaluate dietary patterns is often limited. In particular, it has been suggested that the notion that fruit and vegetables intake represents the main dietary sources of polyphenols could be over-reported [4]. Finally, as far as polyphenols are concerned, the low bioavailability and extensive metabolism demonstrated in numerous studies makes it difficult to clearly state recommendations on intake.

Nevertheless, the analysis of polyphenol intake data registered in several target population with different dietary patterns and lifestyle/exposure may help better understanding whether it is possible to identify a range of intake apparently associated to an overall reduced risk.

To this aim a comprehensive updated review on data and tools/methods used for the estimation of polyphenol intake was performed by considering differences in total and subclasses intake depending on factors related to dietary habits. In addition, main results on the association among polyphenol intake and specific endpoints of disease risk have been taken into account, when available, to suggest possible recommendation.

### 1.1. Search Strategy and Study Selection

A literature search of all English language studies published was performed using PubMed (http://www.ncbi.nlm.nih.gov/pubmed), and EMBASE (http://www.embase.com/) databases (updated December 2018) with the addition of other scientific papers of relevance fount in web sources or in previously published reviews. The search terms and strategy used for the study selection were: polyphenols *OR* flavonoids *OR* anthocyanins *OR* flavanols *OR* flavanones *OR* flavones *OR* flavonols *OR* isoflavones *OR* proanthocyanidins *OR* phenolic acids *OR* hydroxycinnamic acids *OR* hydroxybenzoic acids *OR* lignans *OR* stilbenes *AND* intake. Human studies were used as further criteria of literature search. The search was limited to the last 10 years of publication. Three independent reviewers (S.B., M.M. and M.T.) conducted the literature search in the scientific databases and assessed and verified the eligibility of the studies based on the title and abstract. Disagreement between reviewers was resolved through consultation with a third reviewer (P.R. or C.D.B.) to reach a consensus. Inclusion criteria: (i) prospective, cohort and case-control studies analysing/estimating dietary total/classes/individual polyphenol intake; (ii) studies reporting association between dietary total/classes/individual polyphenol intake and endpoints of disease risk and mortality; (iii) studies published from January 2008 to December 2018. The exclusion criteria were: (i)-dietary intervention studies; (ii)-studies measuring polyphenols intake through urine excretion; (iii)-studies performed in in-vitro or in animal models; (iv)-studies reporting data on polyphenol intake from supplements (not food related); (v)-studies evaluating the association between polyphenol intake and cancer risk/mortality (numerous systematic reviews and meta-analysis have been recently performed); (vi)-published articles in a language different from English and with no accessible translation.

### 1.2. Data Extraction

For the papers meeting the inclusion criteria, the full text was retrieved, analysed and summarized in Tables. Data extraction was performed by three independent reviewers (S.B., M.M., M.T., P.R. and C.D.B.). The following information was collected: (i) first author name and year of publication; (ii) study design; (iii) number and subjects’ characteristics; (iv) country; (v) tools used for estimating dietary polyphenols intake; (vi) polyphenol database source; (vii) overall results. For the studies evaluating the association with disease risk or mortality this information was included in the table. Additional revisions of contents have been performed by other reviewers (N.H.L., B.K. and B.C.).

## 2. Results

### 2.1. Study Selection

**Figure 2:**
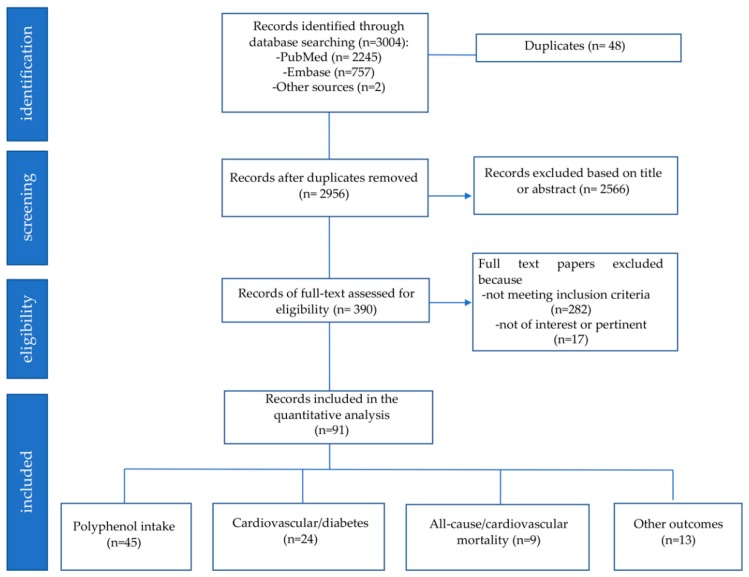
PRISMA Diagram.

The study selection process according to PRISMA guidelines is reported in Figure 2. A total of 3004 records were identified from the database search (PubMed and EMBASE) and other sources. After removing 48 duplicate articles, 2956 studies were screened and 2566 were excluded based on title and/or abstract. The full text of eligible studies (n = 390) was read; 299 studies were excluded because not meeting the inclusion criteria (n = 282) or not of interest/pertinent (n = 17). At the end of the selection process, 91 papers were included.

### 2.2. Study Characteristics

The main characteristics of the 91 included studies are reported in Table 1, Table 2, Table 3 and Table 4; 45 studies focused only on polyphenol intake in specific target populations, 24 studies assessed the association between polyphenols and cardiovascular/diabetes risk (1 study included also data on CV mortality), 9 studies focused specifically on the association with mortality for cardiovascular and all other events, while 13 studies evaluated the association between polyphenol intake with others outcomes (e.g., frailty, bone fractures).

### 2.3. Dietary Intake of Polyphenols

**Figure 3:**
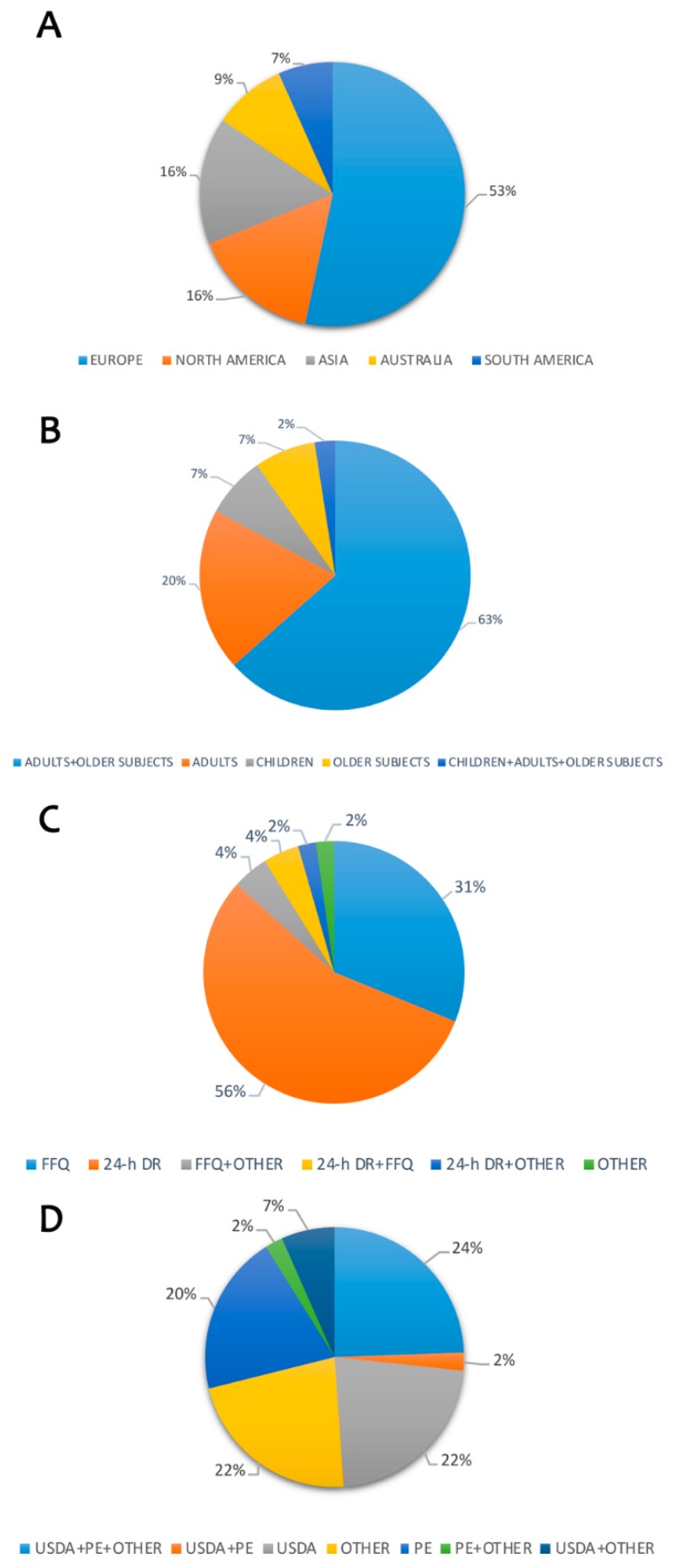
Estimation of polyphenols intake among countries. Legend: (**A**) Target population considered; (**B**) Distribution of published data by country; (**C**) Questionnaires used to evaluate food intake; (**D**) Polyphenol database used for evaluation of intake. FFQ: Food Frequency Questionnaire; 24-h DR: 24-h Dietary Recall; USDA: United States Department of Agriculture; PE: Phenol-Explorer.

Table 1 shows reported data from literature focused on polyphenols intake. A total of 45 studies were found and analyzed [3,4,5,6,7,8,9,10,11,12,13,14,15,16,17,18,19,20,21,22,23,24,25,26,27,28,29,30,31,32,33,34,35,36,37,38,39,40,41,42,43,44,45,46,47]. Most of the studies were performed in Europe, North America and Asia (Figure 3A). The researches (Figure 3B) were carried out in the adult + older population (63%) or only adults (20%), while few studied reported data specifically in older subjects (7%), in children and adolescents (7%); the dietary intake of polyphenols was assessed generally through 24-h dietary records (24-h DR; 56%) and food frequency questionnaire (FFQ; 31%) as reported in Figure 3C. The main scientific databases (Figure 3D) used for the estimation of polyphenol intake were USDA (22%) and Phenol-Explorer (PE; 20%). However, most of the studies combined USDA with PE and other databases and/or scientific sources (24%). Total polyphenol intake for the overall population was estimated to be about 900 mg/day; this value varied according to differences in target groups of subjects. The main food sources of polyphenols were represented by tea, coffee, red wine, fruit and vegetables.

### 2.4. Polyphenol Intake and Cardiovascular Diseases/Diabetes Risk

**Figure 4:**
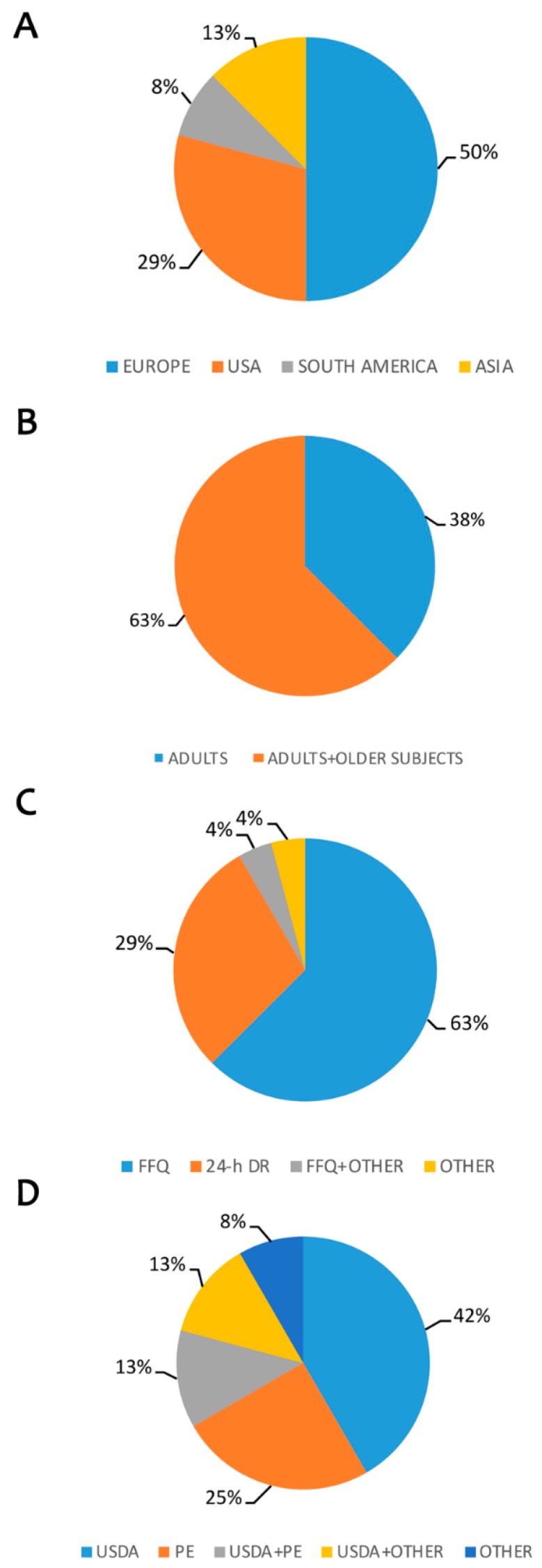
Estimation of polyphenols intake and risk for cardiovascular diseases and diabetes. Legend: (**A**) Distribution of published data by country; (**B**) Target population considered; (**C**) Questionnaires used to evaluate food intake; (**D**) Polyphenol database used for evaluation of intake. FFQ: Food Frequency Questionnaire; 24-h DR: 24-h Dietary Recall; USDA: United States Department of Agriculture; PE: Phenol-Explorer.

In Table 2 the results of studies that examined the association between polyphenol intake and cardiovascular diseases risk are reported [50,51,52,53,54,55,56,57,58,59,60,61,62,63,64,65,66,67,68,69,70,71,72,73]. Seven out of 24 studies were conducted in United States (US), 2 in South America, 12 in Europe, 3 in Asia (Figure 4A). Most of the studies were carried out in the adult population—including older subjects (63%) while the remaining studies were performed in adult population (37%) i.e., aged < 65 years (Figure 4B).

Food intake was mainly assessed through FFQs (63%) or with 24-h DR (29%); 1 study adopted the FFQ in combination with other tools, while 1 study used other assessment methods (Figure 4C).

The main databases used were USDA (42%) and PE (25%). Three studied combined USDA and PE, while the rest of the studies evaluated polyphenol intake with different databases alone or in combination such as Epic Nutrient database, EuroFIR, U.K. Food Standard Agency, Flavonoid Korean Database (Figure 4D).

The association between polyphenol intake and cardiovascular disease risk and diabetes was evaluated by considering several outcomes such as: HDL-cholesterol, triacylglycerols (TAGs), TAG: HDL-cholesterol ratio, HOMA-IR (Homeostatic Model Assessment of Insuline Resistence), Body Mass Index (BMI), cardiovascular events (CV events), stroke events, hypertension and type 2 diabetes (T2D).

On the whole, 12 studies reported an inverse association between polyphenol intake and CV events. In some studies a significant decreased CV risk was observed at the highest quartile of total polyphenol intake (1170 mg/day for Spain and 2632 mg/day for Poland) [57,69] while no effect was demonstrated in other studies performed in Spain and Iran (1248 mg/day and 2459 mg/day respectively) [72,73]. Ten studies evaluated the association with polyphenol subclasses, mainly total flavonoids but only 3 found a significant inverse association with CV events [52,67,72] with intake ranging from 115 to 944 mg/day.

As regard T2D, 1 study performed in Poland showed an increased protection for total polyphenol intake higher than 2632 mg/day while mixed results were found in the other studies focused on total flavonoids and/or subclasses only in some cases able to demonstrate significant T2D risk reduction [53,55,61,62]. Finally, 1 study [67] reported an inverse association for both CV and T2D with the highest quartile of total flavonoids (585 mg/day).

### 2.5. Polyphenols Intake and all-Cause/Cardiovascular Mortality

**Figure 5:**
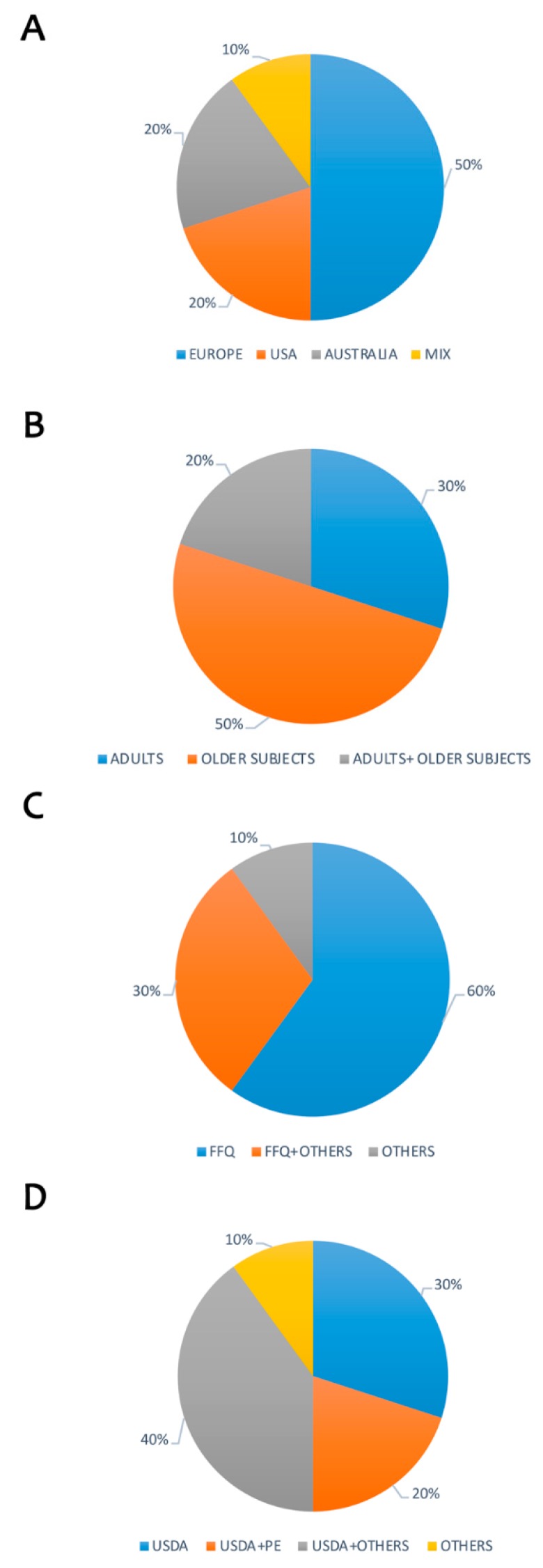
Estimation of polyphenols intake, all-cause and cardiovascular mortality risk. Legend: (**A**) Distribution of published data by country; (**B**) Target population considered; (**C**) Questionnaires used to evaluate food intake; (**D**) Polyphenol database used for evaluation of intake. FFQ: Food Frequency Questionnaire; USDA: United States Department of Agriculture; PE: Phenol-Explorer.

In Table 3 the association between polyphenol intake and all-cause mortality is reported with a specific focus on cardiovascular mortality. A total of 10 studies [59,74,75,76,77,78,79,80,81,82] (Figure 5A) were found; most of the them (50%; 5 out of 10) were performed in Europe (Spain, Italy and The Netherland), 2 in USA, 2 in Australia and 1 was performed including USA, Canada and Australia. Five out of 10 trials (50%) involved older subjects (> 65 years), 3 studies were performed in adults while 2 trails included both adult and older subjects (Figure 5B). The food intake was assessed mainly by FFQ (60%; 6 out of 10 studies); however, some studies (30%) associated FFQs with other tools for the evaluation of food intake (i.e., computerized dietary history questionnaire). One study combined FFQ with EPIC questionnaire (Figure 5C). The evaluation of polyphenol intake was estimated by USDA database (30%; 3 out of 10 studies), or a combination of USDA with others database (40%), or USDA with PE (20%; 2 out of 10 studies). When polyphenol content of specific food-products was missing in available databases, data were obtained from the literature. One study estimated polyphenol intake, in particular monomeric flavan-3-ol, by considering their content in 120 commonly consumed plant foods and beverages obtained by combining results from reverse-phase HPLC and data from literature (Figure 5D).

Overall, one study that investigated the association with total polyphenol intake and all-cause mortality failed to demonstrate a significant effect [75]. Similar findings were also reported by considering the association between total flavonoids and CV mortality [59]. On the contrary, a reduction of mortality risk for cardiovascular events and all–cause mortality was associated with total flavonoid intake in the highest quintiles ranging from 360 mg/day [78] to about 800 mg/day [80]. The impact of the single subclasses has been evaluated in some of the studies, but the effects were conflicting depending on the subject’s characteristics (i.e., age, sex) and cause of mortality. Generally, the models adjusted for the age, as confounding factor, reported a protection also for specific flavonoid subclasses such as isoflavones, flavan-3-ols, flavones. The effects in some cases were found both in women and men. However, generally adjustments for the different confounding factors (i.e., BMI, smoking and alcohol habits, energy intake, physical activity, medications, etc. affected the significance of the associations. 

### 2.6. Polyphenols Intake and other Outcomes

Table 4 shows the associations between polyphenol intake and other outcomes in a total of 13 studies [83,84,85,86,87,88,89,90,91,92,93,94,95]. The associations were evaluated for endothelial function (1 study), kidney function (1 study), bone health (i.e., bone mineral density, frailty and fractures; 3 studies), eyes health (i.e., cataract and macular degeneration; 2 studies), physical performance decline (1 study), dementia (1 study), cognitive decline (1 study) and pubertal development (1 study).

**Figure 6:**
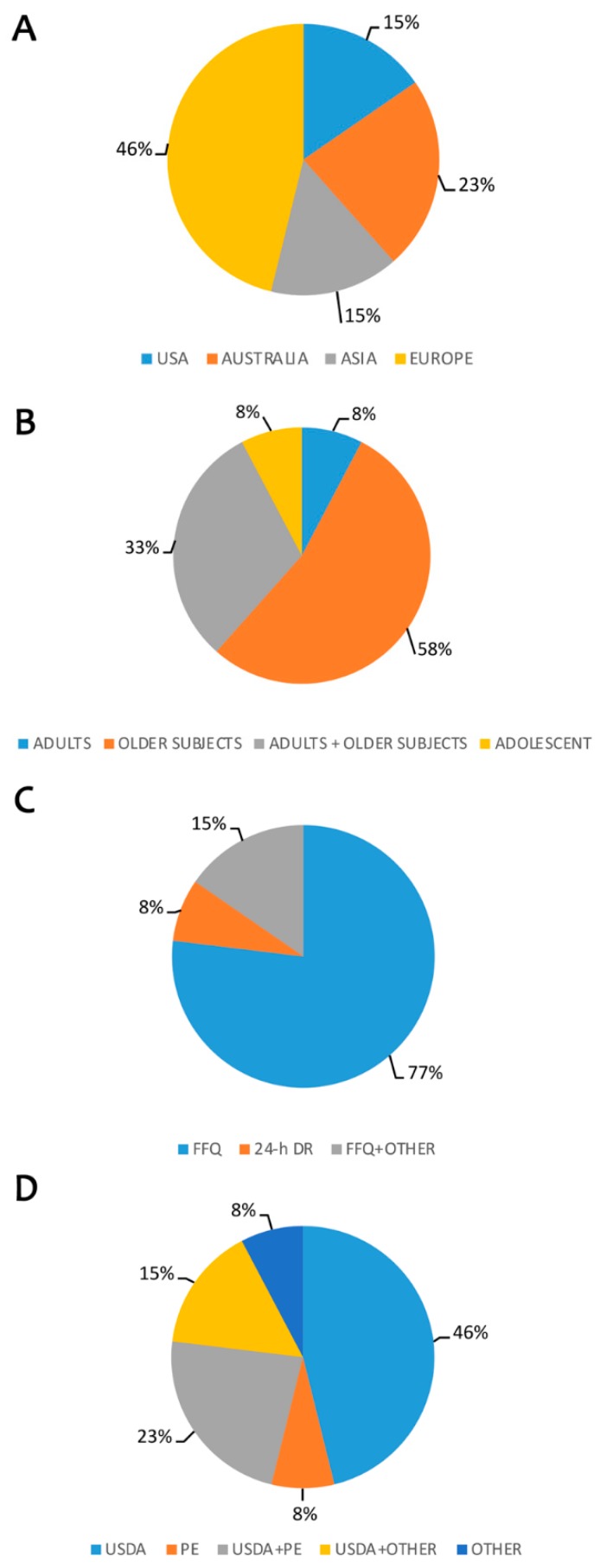
Estimation of polyphenols intake and other outcomes. Legend: (**A**) Distribution of published data by country; (**B**) Target population considered; (**C**) Questionnaires used to evaluate food intake; (**D**) Polyphenol database used for evaluation of intake. Legend: FFQ: Food Frequency Questionnaire; 24-h DR: 24-h Dietary Recall; USDA: United States Department of Agriculture; PE: Phenol-Explorer.

Six out of 13 studies (46%) were performed in Europe, 3 in Australia, 2 in the USA and in Asia Figure 6A). Over than a half of the studies (58%) were carried out in the older population while 33% included adult and older subjects. 1 study was performed only in adults and 1 in adolescents (Figure 6B). The most frequent tools used for the evaluation of the diet were the FFQs (77%; 10 studies), 1 study used a 24-h DR while 2 studies combined FFQs with other tools (Figure 6C). Half of the studies (50%) used USDA database, or a combination of USDA with PE (3 studies) or USDA with other databases (2 studies). Only one study performed the estimation using PE, while one study used a different specific database for the calculation of polyphenol intake (Figure 6D). An overall association between high intake of polyphenols and subclasses, and different outcomes was observed. Conversely, in the InCHIANTI study urinary total polyphenols, but not total dietary polyphenols, were associated with a lower probability of frailty or pre-frailty [86] and cognitive decline [95]. Flavonoids have been associated with a higher endothelial function (>640 mg/day) [83], a lower risk of reduced forced vital capacity and spirometric restriction of the lung (≈290 mg/day) [90], a higher bone mineral density (≈490 mg/day). In addition, flavonoids have been inversely associated with bone fractures (≈1500 mg/day) [85,87] and macular degeneration (≈875 mg/day) [91]. Proanthocyanidins (≥229 mg/day) were inversely associated with risk of renal failure events and kidney insufficiency, while isoflavones (>3 mg/day) with a better pubertal development [84,94].

## 3. Discussion

The great interest for the protective role of polyphenols is demonstrated by the rapid increase of publications evaluating the mechanisms of action of these heterogeneous/complex and multi-target compounds, and also by the studies focused on association between polyphenol intake and different diseases or mortality. In particular, the association of both total or polyphenol subclasses with different types of cancer has been largely addressed in recent reviews and meta-analyses even if the effects are often nulls [96,97,98,99,100,101].

The present study analyzed the literature on polyphenol intake assessment *per se* or in relation to CVD, diabetes, other health outcomes or mortality.

As expected, the review of data obtained from different studies underlines a consistent difference in the estimated polyphenol intake which may be attributed to different methodological issues such as the type of tool administered to assess the intake, the database used for the calculation of polyphenol intake and the type of polyphenols under evaluation.

It is well known that dietary intake is difficult to measure, and single methods (i.e., questionnaires) cannot perfectly estimate dietary exposure. This is particularly critical especially for micronutrients and bioactive compounds. FFQs, and sometimes 24-h DR, represent the main tools used within the epidemiological studies to assess dietary intake. They have different characteristics; for example, FFQs consist in a pre-finite list of foods and beverages (the number of items queried typically ranges from 80 to 120) with response categories to indicate usual frequency of consumption over the time period queried. Conversely, the 24-h DR consists of an open-ended questionnaire administered by a trained interviewer able to collect detailed information about all foods and beverages consumed by the subjects in the previous 24 h. Both questionnaires present several limitations; for example, FFQs lack of detailed information about food preparation, specific food and beverages consumed, as well as different brands. Moreover, the pre-specified food list does not necessarily reflect the eating behavior of the population under study and the presence of systematic errors must be partially mitigated through appropriate statistical modeling that take into consideration the adjustments for cofounding factors such, as an example, age and energy intake. Regarding 24-h DR it requires multiple days to assess usual intake. In addition, multiple administrations are also recommended when 24-h DRs are used to examine diet impact on health outcomes or other parameters. On the other hand, it has been reported that the assessment of total flavonoid intake requires at least 6 days of weighed food records, and between 6 and 10 days to determine intake of specific flavonoid subclasses with an acceptable degree of accuracy [47]. Most of the studies analyzed in the present review did not perform a multiple evaluation of food intake as highly recommended thus, an under or overestimation of total polyphenols and their classes/subclasses intake cannot be excluded.

Another important critical point for the estimation of polyphenol intake is the choice of the databases. The most commonly used are USDA and Phenol-Explorer. USDA database focuses predominantly on flavonoids as aglycones (anthocyanins, flavanols, flavanones, flavones, flavonols and isoflavones), while Phenol-Explorer, in addition to the above mentioned flavonoids (mainly as glycosides), provides data also of the precursors (chalcones, dihydrochalcones and dihydroflavonols) and information on total polyphenols measured by Folin-Ciocalteu [25]. Despite both data sources are systematically extended to reflect most accurately phenolic contents in food, it is clear that they show several limitations. First of all, since they provide information on different classes of polyphenols, the comparison of the results obtained on the basis of the various data sources may differ. For example, some studies reported that the intake of flavonoids are generally higher when calculated using the USDA databases in relation to the Phenol-Explorer database [102]. In addition, despite they provide information on a wide range of foods, the list does not include all food and polyphenol sources; this represents a critical aspect since missing data have to be found by using different databases and/or by consulting the scientific literature with an increase of risk of bias. Moreover, the effect of seasonality, storage and cooking process is not always considered but certainly, it could represent a critical point. Finally, in view of these issues, it should be remarked that all databases allow only an estimation of dietary polyphenols intake. In this regard, it is noteworthy that databases do not consider non-extractable polyphenols thus contributing to an overall under estimation of intake [103]. This is relevant since these compounds seem to have potential protective properties exerted through gut microbiota metabolites production [104].

In the present review, we found that most of the studies used USDA and Phenol-Explorer databases alone, in combination, or together with other databases and/or data sources (i.e., specific scientific publications). An estimation of polyphenol intake data obtained from reviewed studies using FFQs and from those using 24-h DR, seem to provide comparable results in terms of total polyphenol intake (FFQs 910 mg; 24-h DR 890 mg), total flavonoids (FFQs 360 mg; 24-h DR 380 mg) and total phenolic acids (FFQs 410 mg; 24-h DR 450 mg). In addition, it is noticeable that generally data come from single evaluations instead of multiple evaluations of food intake as recommendable, thus an under or overestimation of polyphenols and/or specific subclasses cannot be excluded.

Polyphenol intake is also affected by intrinsic factors such as the geographical area, the population characteristics in term of age, gender and socio-cultural factors and above all the dietary habits. In this regard, we have found that the intake of total polyphenols is higher in Japan (about 1500 mg/day) compared to European countries and North and South America (about 900 mg/day and 800 mg/day respectively). Within Europe, we found a large variability of intake between countries; Poland and France had the highest intake of total polyphenols (above 1000 mg/day), followed by Italy (about 650 mg/day) and Spain (about 300 mg/day). Conversely, within the EPIC study, Denmark showed the highest intake of total polyphenols (1786 mg/day) while Greece the lowest (584 mg/day) [27].

Regarding total flavonoids, Poland and Australia had the highest intake (about 600 mg/day) while USA and South America the lowest (about 200 and 400 mg/day, respectively) followed by Asia (China and Korea, at about 60 mg/day). Finally, regarding total phenolic acids, France, Poland and Brazil had the highest intake (above 600 mg/day), while USA, Italy and Spain the lowest (about 300 mg/day). These data were also in accordance with the results obtained within EPIC study, which showed a high flavonoid and phenolic acid intake in non-Mediterranean countries [15] associated to different dietary habits. For example, in the North and Central Europe, non-alcoholic beverages, in particular tea and coffee, are the main polyphenol contributors, while in South Europe the main contributors are fruits alcoholic beverages (e.g., red wine). In Asia, such as China and Korea, apples and vegetables seem to be the main polyphenol sources, while green tea in the Japanese population. Finally, tea, citrus and legumes seem to be the main polyphenol contributors in the USA.

As far as gender differences in polyphenol intake are concerned, data in literature are not univocal even if more studies suggest a higher intake in females compared to males, above all when standardization for energy intake is taken into account. In addition, differences in polyphenol sources selected seem to be dependent on gender (e.g., higher contribution of fruit and vegetables in females compared to males who are higher consumers of alcoholic beverages and coffee).

Notwithstanding, most of the data available have been assessing polyphenol intake in adults, a large number of studies considered also the intake in older subjects. Nine studies specifically reported results on total polyphenol and/or subclasses in target of older populations (2 Australia, 2 Spain, 1 Brazil, 1 Italy, 1 Poland, 1 UK and 1 Japan). Total polyphenol intake ranged from about 333 mg/day in Spain [44] to 1492 mg/day in Japan [32]. In addition, those considering total flavonoid intake registered values from about 170 mg/day in Spain [44] to about 834 mg/day in Australia [102]. When available the contribution of phenolic acids was approximately 30–40% of the total polyphenol intake. Studies considering different age classes found controversial results, even if generally, all studies reported differences in food habits affecting polyphenol intake. For example, Vitale et al. [48] showed that flavonoid and stilbene increased with age in the TOSCA.IT study, being higher in over 65 years subjects compared to those with age lower than 65 years. Accordingly, Miranda et al. [39] reported that older subjects (>60 years) from a Brazil cohort consumed more flavonoids and tyrosol than adults (20–59 years) and also more fruits. Moreover, Zamora-Ros et al. [27], showed an increased intake of flavonoids, stilbenes, lignans and other polyphenols with age, while no effect on total polyphenol intake in the EPIC cohort. Other studies reported no differences in polyphenol intake depending on age, or a slight increase after energy adjustment [43,49]. Others (Zujko et al. [19]) showed lower levels of flavonoid intake in older Brazilian subjects who generally consumed less beverages and vegetables. Finally, Karam et al. [44] found an increased energy adjusted polyphenol intake by age classes in older adults from Mallorca island showing also the impact of factors such as gender, educational level and lifestyle significantly affecting eating habits. Large differences in food selection depending on region/country have been underlined reflecting a different pattern of polyphenol intake.

Only 3 studies reported data on children and adolescents showing a low polyphenol intake associated to the overall dietary pattern generally poor in fruit and vegetables even if direct comparison among results is difficult due to the lack of energy adjustment of data in the different age subclasses. The main sources of polyphenols identified depending on the country were non-alcoholic beverages (UK, Argentine), fruit (apple, pear), juices, chocolate (in Helena European study [46]).

Extensive research on polyphenols in human studies has shown a potential role of these compounds in the modulation of CVD markers [105]. In the present systematic review, we found an overall inverse association between total polyphenol intake (highest quantile, above 1170 mg/day) and CV risk events and mortality. In addition, an increased protection against T2D events was observed for total polyphenol intake (mean intake of the 4th quartile) higher than 2632 mg/day [69]. However, the results are not univocal and 4 out of 9 papers reported no association at doses of polyphenols higher than 1200 mg/day or above (>2400 mg/day). These conflicting results could be attributed to the high heterogeneity of the studies in term of selected population characteristic, markers/endpoints measured (i.e., marker of CV risk analyzed), dietary habits (very different between countries), and polyphenol food sources (i.e., tea, coffee, fruits, alcoholic beverages).

Recent evidence from systematic reviews and meta-analyses of cross-sectional and prospective cohort studies seem to suggest that the intake of certain polyphenol classes and subclasses, more than total polyphenols, may reduce the incidence of T2D, CVD events and CVD mortality. However, most of the effects were found when comparing the highest quantiles *versus* the lowest. In fact, we reported a lower risk of CV events for an intake of total flavonoids ranging from 115 to 944 mg/day, an inverse association for T2D with the highest quartile of total flavonoids (585 mg/day), and a low risk of mortality for cardiovascular events and all–cause mortality for the highest quintile of total flavonoid intake (range 360–800 mg/day) [78,80]. These results are in line with observations reported by other authors. For example, McCullough et al. [74], showed that a total flavonoid intake above 512 mg/day was inversely associated with fatal events for CVD in men and women. Feliciano and coworkers [106], reported that high consumers (>788 mg/day of total flavonoids) showed an inverse association with CVD events and CVD mortality. Wang and colleagues [107] found a reduced risk of CVD events for doses of flavonoids (including flavonols, anthocyanidins, proanthocyanidins, flavones, flavanones and flavan-3-ols) between 139 and 604 mg/day. Finally, Grosso et al. [108] showed that increasing by 100-mg/day flavonoid intake led to a linear decreased risk of 6% and 4% of all-cause and CVD mortality.

As regard the diverse subclasses of polyphenols, several studies have reported a positive effect for flavonols, flavones, flavanones, isoflavones, anthocyanidins and proanthocyanidins. For example, Wedick and coworkers [53], have shown that the highest quintile of anthocyanins (about 22.3 mg/day) and anthocyanin-rich fruit intake (≥5 times/week) was associated with a lower risk of T2D. Conversely, limited evidence is available for lignans. One study performed by Rienks and colleagues [109] showed that high levels of plasma enterolactones (lignan precursors) were associated with a 30% and 45% reduction of all-cause and CVD mortality risk.

Interestingly, in the last years, a growing attention has been devoted to the impact of polyphenols on different health outcomes including for instance renal insufficiency, respiratory function, immune function, and vascular activity. For these outcomes, flavonoids and proantocyanidins have shown an apparent promising beneficial effect. Very recently, another research path has focused on the contribution of polyphenols in the older subject health outcomes. Specifically, the effect on retardation/prevention of some age-related complications such as cognitive decline, frailty and bone fractures has been investigated. On the whole, we have found an overall positive association between high intake of polyphenols and classes/subclasses, and a modulation of different outcomes associated with aging. In particular, total flavonoids and subclasses have been apparently associated with a higher bone mineral density, low risk of bone fractures and macular degeneration, while only total urinary polyphenols, but not dietary polyphenols, have been associated with a low risk of pre-frailty and frailty in older subjects. However, this type of investigation is at early stages thus, further studies have to be performed in order to strength the evidence on the associations found. In addition, since the preliminary observations on protective effects have been found mainly for specific compounds, future studies should be focused on the contribution of subclasses or individual polyphenolic compounds, and even metabolites, instead of total polyphenols.

## 4. Conclusions

Undoubtedly, polyphenols exert numerous biological activities as reported in a plethora of in vitro and in vivo studies. In addition, several systematic reviews and meta-analyses of observational and intervention studies have found a reduced risk for numerous chronic diseases. We documented an overall inverse association between polyphenol intake and CV risk events and mortality, as well as, between polyphenols and other outcomes of health status. However, most of the associations were found for specific polyphenol classes/subclasses as well as markers/endpoints. At present, few and conflicting results are available for total polyphenols thus, as also reported more than 10 years ago [110], it is still difficult to establish a reference and/or prudent intake of total polyphenols, even if we found an approximate mean intake of about 900 mg/day. Some studies suggest an inverse association between high total flavonoid intake (generally higher 500 mg/day) and CV events and/or mortality. However, this value should be considered as a temptative level due to the elevated heterogeneity of the studies and the numerous limitations associated with the evaluation and estimation of polyphenol intake. It is then fundamental to consider that polyphenol intake correspond to differences in dietary behavior and selection of diverse food sources of the same compounds could affect the overall impact differently. Therefore, it is reasonable to argue in terms of dietary patterns more than focusing on single contributions. In this context, polyphenol-rich dietary pattern seems to exert health benefits and should be considered a valid tool for the prevention of numerous chronic diseases.

At the same time, further investigation is highly recommended in order to address the need for: (1) improved dietary assessment methods; (2) standardized and validated analytical procedures for the analysis of polyphenols and related subclasses in foods; (3) implementation of food databases increasing food items and information available on the different polyphenol subclasses; (4) validation of specific polyphenol intake biomarkers. Nevertheless, despite information from observational studies are necessary to identify potential role of diet-related compounds, the availability of well controlled and specifically targeted dietary intervention studies (addressing also dose-response effects) seems to be mandatory to allow the identification of a reference or prudent intake (e.g., in term of health-promoting properties) for food bioactives such as polyphenols, directed to the general population or specific vulnerable groups (e.g., older subjects).

## Figures and Tables

**Table 1:** Polyphenol intake registered in adults.

Reference by Year	Population Characteristics	Country	Dietary Assessment n° Food Containing Items	Polyphenol Database n° Food Items	Estimated Intake (mg/day) mean/median/min-max	Polyphenol Main Subclasses Intake (mg/day or percentage) mean ± ds/median/min-max	Main Dietary Sources (Based on % Contribution)	Overall Results
Song et al. [5]	8809 subjects (NHANES 1999–2000 and 2001–2002) W = 4348 M = 4461 Age = >19 year	US	1 24-h DR	USDA Database ^(1,2)^	**Total flavonoids** Mean intake = 189.7 ± 11.6	**Flavan3-ols** Mean intake = 156.5 ± 11.3 **Flavanones** Mean intake = 14.4 ± 0.6 **Flavanols** Mean intake = 12.9 ± 0.4 **Anthocyanidins** Mean intake = 3.1 ± 0.5 **Flavones** Mean intake = 1.6 ± 0.2 **Isoflavones** Mean intake = 1.2 ± 0.2	Tea (82.8%) Citrus juices (4.3%) Wine (2.1%) Citrus fruits (1.8%)	Different total flavonoids intake was observed between tea consumers (21% of the population) and tea non-consumers (697.9 vs. 32.6 mg/day respectively) with flavonols and flavan-3-ols as main compounds
Ilow et al. [6]	203 subjects W = 121 M = 82 Age = 50 year	Poland	FFQs 48 food items	USDA database ^(1)^	**Total flavonoids** (median) M+F = 610.8 M = 612.0 F = 609.2	n.a.	Tea Fruit Vegetable	The flavonoid intake in tea was the same in women as in men. Tea flavonoids constituted about 96% of all the consumed flavonoids in this population
Otaki et al. [7]	514 subjects W = all M = 0 Age = 58 ± 10 year	Japan	1 24-h WDR	FFF (functional food factor) database	**Total polyphenols-**	* **Total flavanols** Mean = 1277 ± 1403 * **Total isoflavones** Mean = 215.7 ± 147.3 * **Total flavonols** Mean = 58.4 ± 62.7 * **Total flavanones** Mean = 30.5 ± 145.8 * **Total flavones** Mean = 15 ± 51.6 * data expressed in µmol/d	Green tea Onion Soy processed food (tofu, natto and miso)	The study showed higher total flavonoid intake compared to previous studies performed in the Japanese population. The sources of flavonoids differed from those of Western countries. Green tea, soy foods and onion constituted the main sources of flavan-3-ols, isoflavones and flavonols, respectively. Grapefruits and citrus fruits were the main sources of flavanones, while Malabar spinach, green peppers and grapefruits the main sources of flavones
Chun et al. [8]	8809 subjects NHANES 1999–2000 (n = 4175) and 2001–2002 (n = 4634) W = 4348 M = 4461 Age = >19 year	US	1 24-h DR	USDA Database ^(1,2)^	**Total flavonoids (1999–2000)** Mean intake = 209.8 ± 18.9 **Total flavonoids (2001–2002)** Mean intake = 204.5 ± 14.5	n.a.	Tea (76.8%) Citrus fruit juice (3.7%) Beers and ales (2.9%) Wine (2.4%) Citrus fruit (1.7%) Melon and berries (1.4%) Other vegetables (1.4%)	Daily intake of flavonoids was dependent on sociodemographic characteristics and lifestyle behaviors. Daily flavonoid intake was provided mainly by teas (i.e., catechins)
Yang et al. [9]	128 subjects W = n.a. M = n.a. Age = 20–28 year	China	2 sFFQs 126 food items 2 7-day 24-h DRs (used to validate FFQs data)	Specifically developed database *	**Total flavonoids (FFQ1)** Mean intake = 45.39 ± 25.52 **Total flavonoids (FFQ2)** Mean intake = 46.94 ± 27.72 **Total flavonoids (24-h DRs)** Mean intake = 50.15 ± 35.83	FFQ 1 data: **Total flavonol** Mean intake = 34.74 ± 18.80 **Total flavone** Mean intake = 10.65 ± 7.02 FFQ 2 data: **Total flavonol** Mean intake = 35.75 ± 20.45 **Total flavone** Mean intake = 11.19 ± 7.57 24-h DRs data: **Total flavonol** Mean intake = 38.37 ± 28.59 **Total flavone** Mean intake = 11.78 ± 8.45	n.a.	The FFQ used had reasonable reproducibility (measured 1 year apart) and validity to estimate dietary intake of flavonols (quercetin, kaempferol, isorhamnetin) and flavones (apigenin, luteolin) in the Chinese population, as compared to the other type of assessment methods
Zhang et al. [10]	5046 subjects W = 2910 M = 2136 Age = 18–72 year	China	2 sFFQs 126 food items 2 7-day 24-h DRs (used to validate FFQs data)	Specifically developed database *	**Total flavonols-Flavones** Mean intake = 19.13 ± 8.28	**Flavonols** Mean intake Quercetin = 5.96 ± 3.09 Kaempferol = 4.14 ±1.95 Myricetin = 1.81 ± 1.24 Isorhamnetin = 2.34 ± 1.48 **Flavones** Mean intake Apigenin = 1.06 ± 0.56 Luteolin = 3.82 ± 1.88	Apple (12%) Potato (8%) Celery (7%) Eggplant (7%) Actinidia (5%)	The total intake of flavonols and flavones was higher in men than in women. Gender and above all age were independent predictors for total flavonols and flavones intake. Main food sources were vegetables (61%) and fruits (36%) while tea was only a minor source
Hanna et al. [11]	551 subjects W = 551 M = 0 Age = 40–79 year	Australia	Phytoestrogen frequency questionnaire 112-item	USDA and specific literature	**Total isoflavones-lignans** Mean = 8.44 ± 17.03 Median intake = 2.2 Min and max = 0.44–174	**Total isoflavones** Mean = 4.5 ± 10.07 Median = 0.03 Min and max = 0–98 Total Lignans Mean = 2.71 ± 3.04 Median intake = 1.83 Min and max = 0.16–33	Soy and soy product (tofu, miso, soy grits or cereal)	Isoflavone intake was significantly different depending on age, i.e., 40–49 years and 50–59 years age groups introduced higher isoflavone amount compared to 60–69 years and 70–79 years age groups. There was no significant difference in lignan intake among age groups
Péréz-Jimenéz et al. [12]	4942 subjects (SU.VI.MAX cohort 1994.1995) W = 2346 M = 2596 Age = 45–60 year	France	6 24-h DRs 736 food items	Phenol Explorer	**Total polyphenols** Mean intake = 1193 ± 510 Median intake = 1123	**Flavonoids** Mean intake = 506 ± 219 **Phenolic acids** Mean intake = 639 ± 273	Non-alcoholic beverages (55.2%) Fruit (17.3%) Alcoholic beverages (8.3%) Cocoa products (7.5%) Vegetables (6.8%) Cereals (3.9%)	Total polyphenol intake was higher in men than in women. Age had no significant influence on intake. Three beverages Coffee, tea, and red wine accounted for 44%, 9%, and 6% of the total polyphenol intake while fruit, cocoa products, vegetables, and cereals for 17%, 8%, 7%, and 4% of the total polyphenol intake confirming data from other Western populations
Zamora-Ros et al. [13]	36,037 subjects (EPIC cohort) W = 23,009 M = 13,028 Age = 35–74 year	10 European countries	1 24-h DR (EPIC-SOFT)	USDA database expanded with Phenol Explorer 1877 food items	**Total flavonols-flavanones-flavones** Mean intake ± SEM = 66.76 ± 0.89 W = 70.32 ± 0.65 Min W = 37.2 mg/day (Sweden) Min M = 36.7 mg/day (Sweden) Max W = 97.0 mg/day (UK) Max M = 130.9 mg/day (UK)	**Flavonols** Min = 38.5% (South) Max = 47.4% (North) **Flavanones** Min = 46.6% (UK) Max = 52.9% (South) **Flavones** Min = 5.8% (North) Max = 8.6%. (South)	Citrus fruits Citrus-based juices Tea Wine Fruits Vegetables	A large variation in flavanols, flavanones and flavones intake across European regions was registered Overall, flavanones were the main compounds introduced and UK health-conscious group the highest consumers. The total intake was higher in women and dependent on sociodemographic and lifestyle factors. Main food sources differed being juices and tea intake higher in the north while citrus fruit, juices, vegetables and wine in the south
Wang et al. [14]	8809 subjects NHANES 1999–2000 (n = 4175) and 2001–2002 (n = 4634) W = 4348 M = 4461 Age = >19 year	US	1 24-h DR	USDA Database ^(3)^	**Total proanthocyanidins (1999–2000)** Mean intake (PI) = 88.8 ± 6.3 **Total proanthocyanidins (2001–2002)** Mean intake (PII) = 100.0 ± 4.2	**Monomers** Mean intakePI = 20.9 ± 1.5 PII = 20.7 ± 1.4 **Dimers** Mean intake PI = 15.0 ± 1.0 PII = 15.9 ± 1.1 **Trimers** Mean intake PI = 4.7 ± 0.3 PII = 5.3 ± 0.2 **4–6mers** Mean intake PI = 13.5 ± 1.2 PII = 15.7 ± 0.5 **7–10mers** Mean intake PI = 9.4 ± 0.9 PII = 11.2 ± 0.5 **Polymers** Mean intake PI = 25.4 ± 2.8 PII = 31.4 ± 1.9	Tea Legumes Wines	A south to north gradient intake was observed. In general, a mean intake of 95 mg/day was found represented by polymers (30%), monomers (22%), dimers (16%), 4–6 mers (15%), 7–10 mers (11%), and trimers (5%). After adjustment for energy intake, the PA intake increased with age, in women and in alcohol consumer. Tea, legumes, and wines, contributed to about 48% of daily PA intake
Knaze et al. [15]	36,037 subjects (EPIC cohort) W = 23,009 M = 13,028 Age = 35–74 year	10 European countries	1 24-h DR (EPIC-SOFT)	USDA database Phenol Explorer 1877 food items	**Total flavan-3-ols** Mean intake ± SE MED countries = 268.8 ± 2.6 Non-MED countries = 274.7 ± 1.9 UK = 406.6 ± 7.6 **Total monomers** Mean intake ± SE MED countries = 90.2 ± 0.7 UK = 182.4 ± 3.0 **Total proanthocyanidins** (Mean intake ± SE MED countries = 217.2 ± 2.2 Non-MED countries = 177.9 ± 1.5 UK = 198.4 ± 6.3 **Total theaflavins** (Mean intake ± SE) MED countries = 1.6 ± 0.1 Non-MED countries = 6.5 ± 0.1 UK = 25.9 ± 0.3	**Flavan-3-ols subclasses:** **Flavan-3-ol monomers** MED 18.6% non-MED 32.9% UK 44.9% **PA or condensed tannins** MED 80.8% non-MED 64.8% UK 48.8%; **Theaflavins** MED 0.6% non-MED 2.4% UK 6.4%	Tea Wine Fruits Pulses (UK)	Socio-demographic, anthropometric and lifestyle factors were associated with consumption of flavan-3-ols, PA and theaflavins. Differences among different countries were observed. Flavan-3-ol intake in the UK (health-conscious) was about 2-fold that of the MED countries and mainly due to tea providing theaflavins and epigallocatechins. Overall PA intake was higher in the MED countries, even if with large differences, and non-citrus fruit (i.e., apples and pears) and wine the main sources
Zamora-Ros et al. [16]	36,037 subjects (EPIC cohort) W = 23,009 M = 13,028 Age = 35–74 year	10 European countries of EPIC cohort	1 24-h DR (EPIC-SOFT)	USDA database expanded with Phenol Explorer 1877 food items	**Total anthocyanidin** W: Mean ± SE = 33.52 ± 0.39 Max intake = 44.08 (Turin, Italy) Min intake = 18.73 (Granada, Spain) M: Mean ± SE = 29.44 ± 0.53 Max intake = 64.88 (Turin, Italy) Min intake = 19.83 (Bilthoven, The Netherlands)	**Cyanidin** Mean intake W = 15.09 ± 0.23 M = 12.01 ± 0.31 **Delphinidin** Mean intake W = 2.71 ± 0.09 M = 2.26 ± 0.13 **Malvidin** Mean intake W = 9.94 ± 0.18 M = 10.27 ± 0.25 **Pelargonidin** Mean intake W = 3.02 ± 0.09 M = 2.19 ± 0.12 **Peonidin** Mean intake W = 1.64 ± 0.04 M = 1.49 ± 0.05 **Petunidin** Mean intake W = 1.13 ± 0.02 M = 1.23 ± 0.03	Fruits, nuts and seeds (38.1–61.2%) Wines (14.4–24.5%) Non-alcoholic beverages (15.8%) Vegetables (4.8–9.7%)	The highest total anthocyanidins (mainly cyanidins and malvidins). intake was recorded in the south European region. Women (central- southern regions) were the highest consumers. Main food sources were different depending on countries. Central and northern countries: non-citrus fruits (berries, apples and pears, and grapes), wine and non-alcoholic beverages (juices and soft drinks of anthocyanidin-rich fruits). Southern countries: wine, non-citrus fruits (grapes, stone fruits, apples and pears, and olives) and leafy vegetable. A possible underestimation of anthocyanidin intake have been hypothesized due to missing food composition data
Beking et al. [17]	Subjects = n.a.	UK Ireland	FAO Food Balance Sheets	USDA Database ^(1)^ Lacking data from literature	**Total flavonoids** Mean intake Ireland = 176.8 UK = 182.2	Ireland (mean intake): **Anthocyanidins** = 60.3 **Flavanols** = 47.4 **Flavanones** = 29.0 **Flavones** = 5.8 **Flavonols** = 34.2 UK (mean intake): **Anthocyanidins** = 69.2 **Flavanols** = 52.4 **Flavanones** = 26.0 **Flavones** = 4.0 **Flavonols** = 30.3	Grapes and oranges (41.6% UK, 34.9% Ireland) Beer and wine (8.8% UK, 12.8% Ireland) Apples and onions (6.8% UK, 6.5% Ireland) Tea (4.0% UK, 5.3% Ireland).	Estimated dietary intake of anthocyanidins, flavanones, flavanols, flavonols, flavones, and all five combined is similar in the UK and Ireland. Anthocyanidins and flavanols were about 65% of total intake. Data on flavones and flavonols were in line with those obtained in food intake surveys in UK and US. In general, as more types of food flavonoids are analyzed and included in food composition databases, intake estimates are expected to rise and to be more accurate
Ilow et al. [18]	1520 subjects Cardiovascular Disease Prevention Program) W = 879 M = 641 Age = 49–50 year	Poland	FFQs 1 24-h DR	USDA Database ^(1)^	**Total flavonoids** Mean intake W = 622.6 M = 616.9	**Flavan-3-ols** W = 93.6% of total flavonoid M = 94.2% **Flavonols** W = 4.0% M = 4.2% **Anthocyanidins** W = 0.9% M = 1.1% **Flavanones** W = 0.9% M = 0.9% **Flavones** W = 0.1% M = 0.1%	Tea (93.6%, 94.2%) Fruits (2.2%, 1.6%) Vegetables (1.4%, 1.1%) Fruit juices (0.7%, 0.8%) Chocolate (0.1%, 0.1%)	A higher flavonoid intake was reported in comparison with other studies. Tea was the main food source of total flavonoids and mainly of flavan-3-ols intake (from tea, fruits, fruit juices, chocolate)
Zujko et al. [19]	6661 subjects (Polish National Multicenter Health Survey, WOBASZ) W = 3529 M = 3132 Age = 20–74 year	Poland	1 24-h DR	Database of polyphenol contents in food products (developed by the authors) 118 items	**Total polyphenols** Mean intake W = 10,311,054 (20–40 years) 1089 (41–60 years) 947 (61–74 years) M = 1172 1251 (20–40 years) 1183 (41–60 years) 1076 (61–74 years)	n.a.	Beverages (tea, coffee) Vegetables (potato) Fruits (apples) Cereals (white bread)	Polyphenol intake was about 1 g independently from gender and age and apparently similar to that of other countries. However, patterns of consumption were different depending on gender and age groups
Lee et al. [20]	8502 subjects W = n.a. M = n.a. Age = >2 year	Korea	1 24-h DR	Phytonutrient database (Korea National Academy of Agricultural Science)	**Total polyphenols-**	Subjects meeting the recommendations **Anthocyanidins** = 73 ± 4.8 **Hesperitin** = 25.4 ± 3.2 **Catechin** = 24.8 ± 1.4 **Quercetin** = 9.1 ± 0.3 **Isoflavones** = 25.8 ± 2.8 **Gallic Acid** = 18.9 ± 2.6 Subjects not meeting the recommendations: **Anthocyanidins** = 8.7 ± 0.3 **Hesperitin** = 3.5 ± 0.5 **Catechin** = 2.2 ± 0.2 **Quercetin** = 2.9 ± 0.1 **Isoflavones** = 5.4 ± 0.5 **Gallic acid** = 4.3 ± 0.7	Fruits Onions Soybeans Nuts	Flavonoids (anthocyanidins, hesperitin, quercetin, catechin, and isoflavones), and one phenolic compound (gallic acid) were significantly higher among subjects who met the recommendations for fruit and vegetable consumption compared with those who did not
Zamora-Ros et al. [21]	36,037 subjects (EPIC cohort) W = 23,009 M = 13,028 Age = 35–74 year	10 European countries	1 24-h DR (EPIC-Soft)	USDA database ^(1)^	**Total polyphenols** **-**	**Total thearubigins** M: Min = 0.9 Max = 532.5 W: Min = 1.2 Max = 455.6	Tea	Large differences in dietary thearubigins (TR) estimations intake across European countries; TR intake is low in Spanish men and high in men from UK; TR contributed < 5% to the total flavonoid intake in Greece, Spain and Italy while contributed 48% to the total flavonoids intake in UK
Tresserra-Rimbau et al. [22]	7200 subjects (PREDIMED) W = n.a. M = n.a. Age = 55–80 year	Spain	FFQs	Phenol Explorer 137 foods item	**Total polyphenols** Mean intake = 820 ± 323	**Flavonoids** = 443 ± 218 **Phenolic acids** = 304 ± 156 **Other polyphenols** = 71.2 ± 46.7	Fruits (44%) non-alcoholic beverages i.e., coffee (55%), vegetables (12%) alcoholic beverages (10%) Olive oil (11%)	Coffee and fruits resulted the main sources of polyphenols even if olives and olive oil represented significant and peculiar Mediterranean dietary sources of polyphenols (i.e., hydroxycinnamic acids, other phenolic acids, lignans and other polyphenols) with respect to other countries
Vogiatzoglou et al. [23]	15,371 subjects W = 8278 M = 7093 Age = 14–80 year	Germany	1 24-h DR (EPIC-SOFT)	FLAVIOLA Database	**Total polyphenols-**	**Total flavanols** Mean intake = 385.9 Min = 195.8 Max = 840.7 **Proanthocyanidins** Mean intake = 196.4 Min = 138.7 Max = 300.3 **Flavan-3-ol monomers** Mean intake = 119.8 Min = 18.3 Max = 414.3 **Theaflavins** Mean intake = 69.7 Min = 38.8 Max = 126.1	Data are referred to total flavanols: Pome fruits (27%) Black tea (25%) Non-alcoholic beverages (46%) Green/fruit herbal tea (10–16%) Berries (6%)	Women had slightly higher intakes of total flavanols than men in all age groups, except for the elderly. There was a steep age gradient with an increase in total flavanols, flavan-3-ol monomers, and theaflavins across the age groups. Proanthocyanidins were the main contributor of total flavanols in both men and women
Grosso et al. [24]	10,477 subjects (HAPIEE study) W = 5340 M = 5137 Age = 45–69 year	Poland	FFQs 148 items	Phenol Explorer	**Total polyphenols** Mean intake = 1740.7 ± 630.2 Median intake = 1662.5	**Total flavonoids** Mean intake = 897.6 ± 423.4 **Total phenolic acids** Mean intake = 800.2 ± 345.8	Coffee (40%) Tea (27%) Chocolate (8%)	Intakes were slightly higher in men than in women, but when adjusted for energy intake, women had a higher intake of polyphenols than men. Age had significant influence on total and energy-adjusted polyphenol intake, being higher among younger participants
Witkowska et al. [25]	6661 subjects W = 3529 M = 3132 Age = 20–74 year	Poland	24-h DR	Phenol Explorer USDA database ^(1–3)^	**Total polyphenols** Mean Intake = 989.3 ± 360	**Total flavonoids** Mean Intake USDA = 524.6 ± 155 PE = 403.5 ± 150 **Total phenolic acids** Mean Intake USDA = n.a. PE = 556.3 ± 204	Total polyphenols (PE): Non-alcoholic beverages (75%) Total flavonoids: Non-alcoholic beverages: (PE 78.5%) (USDA 90%)	Flavonoids estimated through various databases might substantially differ. The use of several databases can truly reflect the real intake but it will be difficult to comparison for which only one method has been used for calculations
Kim et al. [26]	11,474 Subjects W = n.a. M = n.a. Age = ≥ 19 year	Korea	1 24-h DR	USDA database ^(1)^ Korean-targeted flavonoid database	**Total flavonoids** Mean Intake ± SE = 96.6 ± 1.34 Median = 70.4 P10 – P90 = 22.8 – 192	**Total anthocyanidins** Mean Intake ± SE = 26.4 ± 0.9 Median = 6.36 P10 – P90 = 0 – 68.1 **Total flavanols** Mean Intake ± SE = 25.5 ± 1.8 Median = 1.08 P10 – P90 = 0 – 43.2 **Total flavanones** Mean Intake ± SE = 8.15 ± 0.39 Median = 0P10 – P90 = 0 – 25.1 **Total flavones** Mean Intake ± SE = 0.87 ± 0.03 Median = 0.45 P10 – P90 = 0.13 – 1.86 **Total flavonols** Mean Intake ± SE = 24.6 ± 0.42 Median = 16.8 P10 – P90 = 4.88 – 50.2 **Total isoflavones** Mean Intake ± SE = 21.9 ± 0.39 Median = 12.1 P10 – P90 = 0.27 – 53.9	Kimchi (traditional fermented vegetable product) (12%) Green tea (9%) Persimmon (7%) Soybean (7%) Onion (7%) Tofu (6%) Radish (5%) Tangerine (5%) Apple (4%) Pear (3%)	Total Flavonoid intake was lower in Korea than in western countries. A major difference came from tea intake and also by the lower flavonoid density of major sources (kimchi, persimmon, tangerine, onion, radish etc.) in Korea than those (tea, citrus fruit, apples, pears, wine, etc.) in western countries. Contrast the isoflavone intake was much higher than the estimates for western countries due to high intakes of soybeans, tofu, and fermented soy pastes
Zamora-Ros et al. [27]	36,037 Subjects W = 23,009 M = 13,028 Age = 35–74 year	10 European countries of EPIC cohort	1 24-h DR	Phenol Explorer	**Total polyphenols** Mean intake ± SEW = 1192 ± 6 M = 1177 ± 8 highest in Denmark M = 1786 W = 1626 lowest in Greece M = 744 W = 584	**Total flavonoids:** Mean intake ± SEW = 546 ± 4 M = 492 ± 5 **Total phenolic acids** Mean intake ± SEW = 625 ± 6 M = 593 ± 5 **Total lignans** Mean intake ± SEW = 3.6 ± 0.1 M = 2.5 ± 0.2 **Total stilbenes** Mean intake ± SEW = 2.4 ± 0.0 M = 3.0 ± 0.1	MED countries: Coffee (36%) Fruits (25%) Wine (10%) Non-MED countries: Coffee (41%) Tea (17%) Fruits (13%)	Mean intake of polyphenols was three times higher in men from Denmark than in women from Greece. Stratifying by region, mean of total polyphenols intake was in non-MED countries due to the higher intake of phenolic acids. The study showed a large heterogeneity in both the nature of polyphenols and levels of intake across the countries due to different habits and socio-demographics status
Vogiatzoglou et al. [28]	30,000 subjects W = n.a M = n.a Age = 18–64 year	14 Countries	2–7 24-h DR	FLAVIOLA Database	**Total flavonoids:** Mean Intake = 428 ± 49 central region = 506 ± 75 northern region = 348 ± 20 southern region = 301 ± 27 Median Intake = 164 ± 55 central region = 249 ± 87 northern region = 56 ± 22 southern region = 47 ± 7	**Theaflavins and thearubigins** Mean intake = 168 ± 39 Median intake = 89 ± 38 **Proanthocyanidins:** Mean intake = 124 ± 7 Median intake = 27 ± 5 **(Epi)catechin** Mean intake = 24 ± 2Median intake = 7 ± 2 **Gallated compounds** Mean intake = 53 ± 12 Median intake = 28 ± 12 **Anthocyanidins** Mean intake = 19 ± 2 Median intake = 3 ± 1 **Flavonols** Mean intake = 23 ± 2 Median intake = 8 ± 2 **Flavanones** Mean intake = 14 ± 2 Median intake = 1 ± 0 **Flavones** Mean intake = 4 ± 1 Median intake = 1 ± 0 **Flavonoids (monomeric)** Mean intake = 136 ± 14 Median intake = 49 ± 15	Non-alcoholic beverages Fruits	Large regional differences, both in the type of flavonoids consumed and the distribution of intake. Intakes of anthocyanidins (in particular cyanidin) and flavanones (in particular hesperetin) were highest in the Northern Region, in particular in Finland. Within the Central Region, there was also a large variability of intake between countries. While overall flavonoid intake in Ireland was the highest in Europe, the intake of anthocyanidins was the lowest overall, and intake of flavanones was also very low. France was included in the Southern Region as dietary intake was more comparable with intake in Italy and Spain. However, there are some important differences, and the intake of flavan-3-ols and anthocyanidins in France is considerably higher than in the other countries of the Southern Region
Sebastian et al. [29]	5420 subjects W = 2758 M = 2662 Age = >20 year	USA	1 24-h DR	USDA database ^(1)^	**Total flavonoids** Mean intake = 251 ± 16.8 IQR = 18.8–272 W Mean intake = 241 ± 15.2 IQR = 16.3–272 M Mean intake = 263 ± 20.4 IQR = 20.4–271	Mean intake: **Total flavonols** = 19.4 ± 0.91 IQR = 6.05–25.4 **Total flavones** = 0.9 ± 0.1 IQR = 0.1–1.1 **Total flavanones** = 13.1 ± 0.88 IQR = 0.00–5.15 **Total isoflavones** = 1.7 ± 0.3 IQR = 0–0 **Total flavanols** = 204 ± 15.6 IQR = 3.07–189 **Total anthocyanidins** = 11.6 ± 1.07 IQR = 0–9.92	Tea (80%) Fruit Vegetables	A positive association between flavonoid intake and dietary quality suggest that a diet high in flavonoids is synonymous with greater compliance with national guidance. Individuals with higher flavonoids intake not only consume more fruit and vegetables but also eat more healthfully
Kozłowska et al. [30]	151 subjects Polish = 91 Spanish = 60 Polish W = 74 M = 17 Spanish W = 36 M = 24 Total: W = 110 M = 41 Age = n.a.	PolandSpain	FFQs	USDA Database ^(1)^	**Total flavonoids** Mean intake Polish students = 801 Spanish students = 297	n.a.	Polish Students: Black and green tea Oranges Orange juice Spanish Students: Oranges Green tea Orange Juice	Flavonoid consumption in Polish students was more than two times higher than in the Spanish students. The main sources of flavonoids in Spanish and Polish diets were different as black tea in the Spanish group provided weekly about 236 mg of flavonoids, over 12 times less than in the Polish group. On the other hand, the Spanish diet was richer than the polish diet in sources of flavonoids such as oranges, chickpeas, dried parsley, onions, strawberries, almonds or pomelo
Zujko et al. [31]	6661 subjects M = 3132 W = 3529 Age = 20–74 year	Poland	1 24-h DR	Database developed by the authors	**Total flavonoids** Mean intake = 276 **W (20–40 year)** = 278 CI95% = 266–290 **M (20–40 year)** = 304CI95% = 291–317 **W (41–60 year)** = 275 CI95% = 264–286 **M (41–60 year)** = 291 CI95% = 279–311 **W (61–74 year)** = 238 CI95% = 227–249 **M (61–74 year)** = 268 CI95% = 256–280	n.a.	Beverages (47%) Fruit and fruit jams (27%) Tea (22%) Vegetables (18%) Apples (12%) Coffee (8%)	The consumption of tea, coffee, and apples was associated with the largest contributions to the flavonoid content. In comparison to the young and middle age participants, the elderly consumed less beverages and vegetables with a lower level of flavonoids
Taguchi et al. [32]	610 subjects M = 569 W = 41 Age = 52–89 year	Japan	FFQs	Database developed by the author	**Total polyphenols** Mean intake = 1492 ± 665	n.a.	Coffee (43.2%) Green tea (26.6%)	The present study showed that a population of elderly Japanese (mostly men) consumed higher amounts of polyphenols than previous data in Japanese adults, and coffee and green tea were the largest sources of polyphenols in their daily life
Sun et al. [33]	887 subjects W = 887 Age = 12–18 year	China	FFQs 4 24-h DR	Flavonoids database developed by the authors	**Total flavonoids** Mean intake = 20.60 ± 14.12	**Total flavonol** = 16.29 ± 11.91 **Quercetin** = 5.51 ± 4.00 **Kaempferol** = 5.49 ± 3.68 **Myrucetin** = 2.29 ± 1.84 **Isorhamntin** = 3.00 ± 2.37 **Total flavones** = 4.31 ± 2.21 **Luteolin** = 3.27 ± 1.63 Apigenin = 1.03 ± 0.58	Apple (11.7%) Potatoes (9.9%) Lettuce (7.3%) Oranges (7.0%) Chinese Cabbage (4.7%) Tomatoes (4.2%) Celery (4.2%) Soyabean Sprouts (4.2%) Leeks (3.9%) Aubergine (3.9%)	The dietary flavonoid intakes among female adolescents in the Suihua area were similar to those reported in previous studies. In the present study, apples, potatoes, lettuce, oranges, soyabean sprouts and leeks were the main food sources of flavonols, whereas tomatoes, aubergine, white radishes, celery and sweet potatoes were the main sources of flavones
Kim et al. [34]	9801 subjects W = 5032 M = 4769 Age = >19 year	US	2 24-h DR	USDA databases ^(1,2)^	**Total flavonoids** Mean intake = 200.1 ± 8.9	**Total flavonols** Mean intake = 15.9 ± 0.4 **Total flavones** Mean intake = 1.2 ± 0.1 **Total flavanones** Mean intake = 12.2 ± 0.5 **Total flavanols** Mean intake = 158.4 ± 8.5 **Total anthocyanidins** Mean intake = 11.5 ± 0.7 **Total isoflavones** Mean intake = 0.9 ± 0.1	Tea Citrus fruit juices Berries Citrus fruit Wine Apples	Flavonoid intake increased with age from 19 to 30 years until 50–70 years in both men and women. After adjusting for energy intake, flavonoid density of women was greater than those of men (*p* < 0.0001). The difference of flavonoid density among ethnicity was reduced after adjusting for energy intake. Flavonoid density of alcohol non-consumer was greater than that of alcohol consumer (*p* < 0.05)
Burkholder-Cooley et al. [35]	77,441 subjects W = 50,336 M = 27,105 Age = 57 year	USA Canada	FFQs	Phenol Explorer USDA database ^(1-2)^	**Total polyphenols** Mean intake coffee consumers = 1370 ± 1069 non-coffee consumers = 541 ± 368	**Total flavonoids** Mean intake non-coffee consumer = 305 ± 238 coffee consumer = 273 ± 213 **Total phenolic acids** Mean intake non-coffee consumers = 125 ± 106 coffee consumers = 986 ± 1030	Coffee Fruit Vegetables Fruit juice Legumes (including soya)	Significant differences in mean adjusted total polyphenol intakes were observed between dietary patterns. 34% of the participants reported coffee consumption in the FFQ. In the group of non-coffee consumers vegans reported the highest intake of total polyphenols followed by pesco-vegetarians, lacto-ovo vegetarians, semi- vegetarians and non-vegetarians. In the group of coffee consumers non- vegetarians reporting the highest intakes, followed by vegans, semi-vegetarians, pesco-vegetarians and lacto-ovo-vegetarians
Pounis et al. [36]	14,029 subjects W = 7048 M = 6981 Age = n.a.	Italy	EPIC-FFQs specifically adapted for the Italian population 164 food items	Eurofir-eBASIS USDA database	**Total polyphenols** -	Median intake: **Total flavonols** = 17.0 **Total flavones** = 0.7 **Total flavanones** = 32.4 **Total flavanols** = 51.2 **Total anthocyanidins** = 144 **Total isoflavones** = 23.5 **Total lignans** = 80	Seasonal fruits Citrus fruits Leafy vegetable Grain Root vegetables Onions Garlic	Total energy intake was positively associated with the consumption of all polyphenol classes and sub-classes in both genders. Men or older participants seemed to have higher intakes of most of the polyphenols compared with women or younger participants. No significant sex difference was observed for lignans. Educational level did not account for differences in most of flavonoid and lignan intake among participants. No/former smokers presented higher intake of polyphenols. Participants with higher physical activity level consumed greater quantities of all classes of polyphenols
Ivey et al. [37]	1063 subjects W = 1063 M = 0 Age = >75 year Mean age = 80 ± 3 year	Australia	sFFQs	Phenol Explorer USDA database ^(1–3)^	**Total flavonoids** USDA database ^(1–3)^ Mean intake = 834 ± 394 PE Mean intake = 487 ± 243	**Total flavonols** USDA = 30 ± 17 PE = 104 ± 61 **Total flavanols** USDA = 666 ± 345 PE = 327 ± 179 **Total flavones** USDA = 4 ± 3 PE = 13 ± 7 **Total flavanones** USDA = 40 ± 36 PE = 33 ± 31 **Total anthocyanidins** USDA = 88 ± 77 PE = 11 ± 11	n.a.	The mean flavonol PE intake of the cohort was nearly 350% greater than the flavonol USDA estimate. This difference may be, in part, due to the fact that the PE database provides data for five additional groups of flavonol compounds which were not expressed in USDA. Furthermore, the USDA database does not include the flavonol content data of chocolate
Godos et al. [38]	1937 subjects W = n.a. M = n.a. Age = >18 year	Italy	FFQs 110 food items	Phenol Explorer	**Total polyphenols** Mean intake = 663.7 ± 608.1	**Total flavonoids** Mean intake = 258.7 ± 199.1 **Total flavonols** Mean intake = 57 ± 45.6 **Total flavanols** Mean intake = 93.9 ± 118.2 **Total flavanones** Mean intake = 37.9 ± 42.0 **Total flavones** Mean intake = 8.4 ± 10.2 **Total anthocyanins** Mean intake = 55.4 ± 55.3 **Total isoflavones** Mean intake = 4.0 ± 14.4 **Total phenolic acids** Mean intake = 362.7 ± 516.0 **Total stilbenes** Mean intake = 1.9 ± 3.5 **Total lignans** Mean intake = 2.8 ± 2.6	Nuts (29%) Non-alcoholic beverages (23%) Fruits (20%) Vegetables (15%) Alcoholic beverages (7%)	Compared to other Mediterranean cohorts the main differences with all the other cohorts was the contribution of nuts. In this population nuts were among the main contributors of hydroxybenzoic acids, which in other cohorts were generally provided by tea and red wine.
Miranda et al. [39]	1103 subjects W = 678 M = 425 Age = >20 year	Brazil	1 24-h DR	Phenol Explorer	**Total polyphenols** Mean intake ± SE = 377.5 ± 15.3 Median intake = 300.3 IQR = 154.1–486.9	**Mean ± SE** **Phenolic acids** = 284 ± 15.9 **Hydroxycinnamic acids** = 281.2 ± 15.9 **Hydroxybenzoic acids** = 3.4 ± 0.4 **Flavonoids** = 54.6 ± 3.5 **Flavanones** = 16.1 ± 1.9 **Flavonols** = 14.6 ± 0.9 **Flavanols** = 11.4 ± 0.8 **Anthocyanins** = 6.8 ± 1.1 **Flavones** = 3.6 ± 0.3	Coffee (70.5%) Citrus fruit (4.6%) Tropical fruit (3.4%)	The polyphenol intake was three times lower than the estimated value compared with other countries probably due to sociodemographic differences and food choices. Older subjects (>60 y) consumed more flavonoids and tyrosol than adults (20–59 y) and also more fruits.
Burkholder-Cooley et al. [40]	899 subjects W = 602 M = 297 Age = 58 ± 13.2 year	USA Canada	24-h DR FFQs	Phenol Explorer USDA database ^(1,2)^	**Total polyphenols** FFQs Mean intake = 717 ± 646 24-h DR Mean intake = 402 ± 345	n.a.	Coffee Fruit juice	Beverages and fruit were key contributors to total daily polyphenol intake. Subjects could over-report the frequency of intake of fruit and fruit juice in the FFQ even if a positive correlation with 24-h DR is observed.
Bawaked et al. [41]	3534 subjects W = 2015 M = 1509 Age = 2–24 year	Spain	1 24-h DR	USDA database ^(1)^ Phenol Explorer	**Total Flavonoids** Mean intake = 70.7 ± 84.1 Median intake = 48.1 25th–75th percentile = 19.3–93.1	**Total flavonols** Mean intake = 15.6 ± 30.6 Median intake = 5.9 25th–75th percentile = 1.8–17.2 **Total flavones** Mean intake = 2.2 ± 9.1 Median intake = 0.3 25th–75th percentile = 0.0–1.1 **Total flavanones** Mean intake = 19.7 ± 34.1 Median intake = 0.1 25th–75th percentile = 0.0–28.1 **Total flavan-3-ols** Mean intake = 25.2 ± 47.1 Median intake = 14.1 25th–75th percentile = 4.7–28.1 **Total anthocyanins** Mean intake = 7.7 ± 27.1 Median intake = 0.3 25th–75th percentile = 0.0–4.2 **Total isoflavones** Mean intake = 0.1 ± 1.4 Median intake = 0.0 25th–75th percentile = 0.0–0.0	Fruit (42.8%) Cocoa powder and chocolate (23.5%) Vegetables (spinach, onions, artichokes and lettuce) (22%)	Higher adherence to the Mediterranean diet was correlated with higher flavonoids intake. Fruits were the main source of dietary flavonoids
Zamora-Ros et al. [42]	115,315 subjects W = 115,315 M = 0 Age = >25 year	Mexico	sFFQs 140 food items	Phenol Explorer	**Total polyphenols** Median intake = 694 Min and max = 536 and 750 25th–75th percentile = 413–1103	**Total flavonoids** Median intake = 235 Min and max = 188–270 25th–75th percentile = 141–367 **Total phenolic acid** Median intake = 361 Min and max = 243 and 439 25th–75th percentile = 166–690	Total polyphenol: Coffee (29%) Decaffeinated coffee (19%) Total flavonoids: Apple (19%) Orange and mandarins (13%) Orange juice (12%)	Large heterogeneity in intakes of individual polyphenols among Mexican women, but a moderate heterogeneity across Mexican states. Main food sources were also similar in the different states
Ziauddeen et al. [43]	9374 subjects W = 5075 M = 4299 Children (age < 18 year) = 4636 Adults or older (age > 18 year) = 4738 Age > 1.5 year	UK	4D-FR	Phenol Explorer	**Total polyphenols** Mean intake by age ranges = (1.5–3 year) = 266.6 ± 166.1 (4–10 year) = 388.8 ± 188.8 (11–18 year) = 455.0 ± 263.2 (19–34 year) = 635.9 ± 448.9 (35–49 year) = 846.1 ± 514.1 (50–64 year) = 1053.2 ± 545.3 (65+ year) = 1035.1 ± 544.3	**Total flavonoids** (1.5–3 year) = 212.2 ± 151.7 (4–10 year) = 312.1 ± 170.3 (11–18 year) = 355.4 ± 230.9 (19–34 year) = 433.8 ± 335.1 (35–49 year) = 568.3 ± 398.2 (50–64 year) = 714.5 ± 415.2 (65+ year) = 716.2 ± 404.9 **Phenolic acids** (1.5–3 year) = 54.3 ± 24.8 (4–10 year) = 76.5 ± 43.2 (11–18 year) = 99.6 ± 63.4 (19–34 year) = 201.3 ± 228.5 (35–49 year) = 276.2 ± 232.6 (50–64 year) = 336.7 ± 292.0 (65+ year) = 317.6 ± 297.0 **Stilbenes** (1.5–3 year) = 0.1 ± 0.2 (4–10 year) = 0.1 ± 0.1 (11–18 year) = 0.1 ± 0.4 (19–34 year) = 0.8 ± 2.4 (35–49 year) = 1.6 ± 3.8 (50–64 year) = 1.9 ± 4.1 (65+ year) = 1.3 ± 3	Non-alcoholic beverages Fruits	Polyphenol intake increased with age (*p* < 0.001) and was higher in males with exception of adults aged between 19–34 and 50–64 that showed higher levels in females
Karam et al. [44]	211 subjects W = 112 M = 99 Age = 55–80 year	Spain	2 24-h DR	Phenol Explorer USDA databases pecific literature. (449 food items; 245 polyphenol containing products considered)	**Total polyphenols** Mean intake = 332.7 ± 197.4 Median intake = 299.5 IQR = 250.4 **Energy adjusted** Mean intake = 187.5 ± 100.5 Median intake = 172.9 IQR = 140.3	**Flavonoids** = 170.3 ± 144.4 **Flavanols** = 46.0 ± 57.7 **Flavonols** = 22.7 ± 29.9 **Flavanones** = 30.7 ± 50.6 **Flavones** = 10.7 ± 20.3 **Anthocyanin** = 36.7 ± 61.9 **Dihydrochalcones** = 0.3 ± 1.8 **Isoflavonoids** = 19.3 ± 71.1 **Phenolic acids** = 100.0 ± 130.0 **Lignans** = 7.2 ± 15.6 **Stilbenes** = 2.6 ± 4.4	Total polyphenol: Red wine 17.7% Artichoke 6.2% Soy milk 5.4% Total flavonoids: Red wine 26.8% Soy milk 10.8% Orange 9.5%	Flavonoids were the highest ingested polyphenols in the older population under analysis. Polyphenol intake was generally higher in female (adjusted for energy intake), in subjects aged 64–67 y, in physically active and alcoholic product drinkers
Rossi et al. [45]	241 subjects W = n.a M = n.a Age = 6–12 year	Argentine	sFFQs	Phenol Explorer Lacking data from literature	**Total polyphenols** Mean intake = 412	**Phenolic acid** Mean intake = 310 **Flavonoids** Mean intake = 94.1	Mate (60%) Tea (19%) Coffee (5%) Onion (3%)	Low intake of polyphenols was found in this scholar population of high region of the northwest Argentine due to the very low consumption of fruits and vegetables
Wisnuwardani et al. [46]	2428 subjects (HELENA study) W = 1289 M = 1139 Age = 12.5–17.5 year	Different European countries (Greece, Germany, Belgium, France, Hungary, Italy, Sweden, Austria, Spain)	2 24-h DR	Phenol Explorer	**Total polyphenols** Mean intake = 329 Median intake = 326 Q1 = 167 Q4 = 564 Mean intake by age ranges (12.5–13.99 year) = 346 ± 0.1 (14–14.99 year) = 345 ± 0.2 (15–15.99 year) = 356 ± 0.2 (16–17.49 year) = 396 ± 0.2	**Total flavonoids** (12.5–13.99 year) = 267 ± 0.1 (14–14.99 year) = 256 ± 0.1 (15–15.99 year) = 253 ± 0.1 (16–17.49 year) = 271 ± 0.1 **Phenolic acids** (12.5–13.99 year) = 75 ± 0.1 (14–14.99 year) = 75 ± 0.1 (15–15.99 year) = 85 ± 0.1 (16–17.49 year) = 104 ± 0.1 **Stilbenes** (12.5–13.99 year) = 0.038 ± 0.0 (14–14.99 year) = 0.048 ± 0.0 (15–15.99 year) = 0.046 ± 0.0 (16–17.49 year) = 0.060 ± 0.0 **Lignans** (12.5–13.99 year) = 1.0 ± 0.0 (14–14.99 year) = 1.0 ± 0.0 (15–15.99 year) = 1.1 ± 0.0 (16–17.49 year) = 1.1 ± 0.0	Fruit (apple and pear 16%) (23%) Chocolate products (19.2%) Fruit and vegetable juices (16%)	Total polyphenol intake was lower compared to intake of adults reported in previous studies. Polyphenol intake differed largely among countries. Overall, intake for flavonoids was = 75–76% of total polyphenol, for phenolic acids was = 17–19% of total polyphenol and for stilbenes and lignans was = <1% of total polyphenol.
Kent et al. [47]	79 subjects (The Blue Mountains Eye Study) W = 45 M = 34 Age mean = 70.1 year Age = 60–80 year	Australia	12 24-h DR (weighed)	USDA database ^(1)^	**Total flavonoids** Mean intake = 678.69 ± 498.53 Median intake = 581.84 IQR = 619.58	**Anthocyanins** Mean intake = 6.73 ± 12.7 Median intake = 1.05 IQR = 7.88 **Flavonols** Mean intake = 28.04 ± 33.29 Median intake = 24.06 IQR = 21.21 **Flavones** Mean intake = 1.87 ± 4.78 Median intake = 0.55 IQR = 2.11 **Flavan 3-ols** Mean intake = 596.17 ± 494.95 Median intake = 499.72 IQR = 622.95 **Flavanones** Mean intake = 21.43 ± 61.46 Median intake = 2.15 IQR = 12.14	n.a.	Substantial within-individual variation and between individual variation was documented for both total flavonoid intake and intake of flavonoid subclasses. The within-individual variation was in the range 80–140% while the between individual variation was in the range 60–117%. It is speculated that a minimum of 6-day weighed food records is necessary to obtain a reliable estimate of flavonoid intake.
Vitale et al. [48]	2573 subjects (TOSCA.IT Study) W = n. a. M = n. a. Age = 50–75 year Mean = 62.2 ± 0.1 year	Italy	FFQs (Epic)	USDA ^(1)^ Phenol Explorer Lacking data from literature	**Total polyphenols** Mean intake = 683.3 ± 5.8 Mean intake (mg/1000 Kcal/day) Mean = 376.6 ± 3.2 W = 374.0 ± 4.9 M = 378.7 ± 4.1 Mean intake by geographical area North = 387.4 ± 6.0 Center = 355.2 ± 6.1 South = 381.9 ± 4.5 Mean intake by age <60 year = 367.9 ± 4.7 60–65 year = 376.1 ± 5.8 >65 year = 388.4 ± 6.1	**Total flavonoids** Mean intake = 324.7 ± 4.1 **Phenolic acids** Mean intake = 324.2 ± 3.0 **Lignans** Mean intake = 4.1 ± 0.06 **Stilbenes** Mean intake = 3.5 ± 0.11 **Other polyphenols** Mean intake = 27.0 ± 0.27	Non-alcoholic beverages (coffee 54%, tea 27%), fruits (apple 37%, orange 13%), alcoholic beverages (red wine 93%) and vegetables (artichokes 40%, spinach 20%, onions 18%)	A lower intake of polyphenols has been registered in diabetic subjects compared with other groups, showing a different dietary pattern in this type of Italian population.
Nascimento-Souza et al. [49]	620 subjects W = 330 M = 290 Age = 60–98 years	Brazil	Multiple 24-h DR	Phenol Explorer	**Total polyphenols** Mean intake = 1198.6 ± 693.8 Median = 1052.7 IQR = 740.5–1477.9 Mean intake by sex W Mean intake = 1097.6 ± 616 Median = 949.4 IQR = 692.4–1407.9 M Mean intake = 1313.5 ± 757.3 Median = 1169.2 IQR = 844.7–1610.3 Mean intake by age 60–74 years Mean intake = 1197.8 ± 619.3 Median = 1092.4 IQR = 806.9–1502.9 >75 years Mean intake = 1310.2 ± 699.4 Median = 1186.9 IQR = 818.3–1582.2 **Mean intake energy-adjusted** Mean = 1198.6 ± 591.1 Median = 1102.8 IQR = 817.3–1504.8 Mean intake by sex W Mean intake = 1183.8 ± 545.4 Median = 1097.6 IQR = 816.7–1494.8 M Mean intake = 1215.4 ± 639.8 Median = 1116.0 IQR = 829.5–1537.2 Mean intake by age 60–74 years Mean intake = 1197.8 ± 619.1 Median = 1092.4 IQR = 806.9–1502.9 >75 years Mean intake = 1200.7 ± 522.1 Median = 1143.9 IQR = 858.5–1508.6	**Total flavonoids** Mean intake = 444.7 ± 345.1 **Phenolic acids** Mean intake = 729.5 ± 545.4 **Lignans** Mean intake = 13.6 ± 25.5	Non-alcoholic beverages (coffee 45.8%), beans (32.8%), polenta (1.3%)	The intake of polyphenols was in a range similar to that reported for other populations, in particular European countries, but it differs for the main food contributors (high in beans and polenta, low in fruits and vegetables)

Legend: * Cao J, Zhao XJ, Wu K, Zhang Y, and Zhang YQ: Simultaneous determination of five flavonoid compounds in vegetables and fruits by high performance liquid chromatography. Chinese J Prev Med Inf 7, 525–527,2008. n.a. = not available; 24-h DR = 24 h dietary recall; M = men. W = women; FR = food record; FFQ = food frequency questionnaire. ^(1)^ USDA database (Flavonoids) USDA Database for the Flavonoid Content of Selected Foods, Release 2.1. Internet. 2007 Ref Type: Electronic Citation. ^(2)^ USDA database (isoflavones) U. S. Department of Agriculture. Beltsville: MD: USDA; 2008. Database for the Isoflavone Content of Selected foods. Ref Type: Electronic Citation. ^(3)^ USDA database (proanthocyanidins) USDA Database for the Proanthocyanidin Content of Selected Foods. Internet. 2004 Ref Type: Electronic Citation.

**Table 2:** Polyphenol intake and CVD/Diabetes risk.

References	Type of Study	Population Characteristics	Country	Dietary Assessment - n° food-containing items	Polyphenol Database Source n° Food Items	Estimated Polyphenol Intake (mg/day) mean ± sd/quantile/min-max/IQR	Overall Results/Association with Outcome
Huffman et al. [50]	Cohort study	507 subjects W = 263 M = 244 Age = 43–65 year	USA	FFQs	USDA database ^(1)^	**Total flavonoids** Median intake: without diabetes = 280 (387 IQR) with diabetes = 222 (260 IQR)	↓ LDL associated with higher flavanones intake in the group with diabetes ↓ LDL associated with higher flavan-3-ols, and flavanones intake in the group without diabetes ↓ LDL associated with lower polyflavonoids intake in the group without diabetes ↑ HDL associated with higher anthocyanidins and flavan-3-ols intake in the group without diabetes ↓ HDL associated with lower polyflavonoids intake in the group without diabetes There was no relationship between HDL and flavonoids for the group with diabetes.
Pellegrini et al. [51]	Cross-sectional study	242 subjects W = 91 M = 151 Age = 60 year	Italy	3D-WR	Information provided by specific literature ^a^	**Total lignans** Mean (95%CI) Q1 = 382 (332–433) Q2 = 586 (537–636) Q3 = 788 (739–837) Q4 = 1101 (1051–1152)	Total lignans intake are not associated with vascular inflammation and endothelial dysfunction
Cassidy et al. [52]	Cohort study (from NHS I, NHS II, and from HPFS)	156,957 subjects W = 133,914 M = 23,043 Age = 25–75 year	USA	FFQs	USDA database ^(1–3)^ EuroFIR	**Total flavonoids** NHS I Mean = 358 Q1 = 93 Q5 = 944 NHS II Mean = 413 Q1 = 103 Q5 = 1122 HPFS Mean = 376 Q1 = 115 Q5 = 933	↓ 6% hypertension incidence risk associated with higher total flavonoids’ intake (Q5 vs. Q1; RR = 0.94; 95% CI: 0.90–0.99) in NHS I Total flavonoids’ intake was not significantly associated with the risk of hypertension incidence in NHS II (RR = 1.01; 95% CI: 0.95–1.07) e HPFS (RR = 1.06; 95% CI: 0.97–1.16)
Wedick et al. [53]	Cohort study (from NHS I, NHS II, and from HPFS)	200,894 subjects W = 159,560 M = 41,334 Age = 25–75 year	USA	FFQs 118–131-item	USDA database ^(1)^	**Total flavonoids** NHS I Q1 = 105.2 Q2 = 174.8 Q3 = 249.2 Q4 = 369.1 Q5 = 718.1 NHS II Q1 = 112.1 Q2 = 182.5 Q3 = 256.1 Q4 = 378.4 Q5 = 770.3 HPFS Q1 = 112.5 Q2 = 182.2 Q3 = 251.7 Q4 = 352.9 Q5 = 624.3	↓ 15% type 2 diabetes risk associated with higher total flavonoids’ intake (Q5 vs. Q1; HR = 0.85; 95% CI: 0.79–0.92) in NHS I Total flavonoids’ intake was not significantly associated with the risk of hypertension incidence in NHS II (HR = 0.99; 95% CI: 0.89–1.11) e HPFS (HR = 0.92; 95% CI: 0.81–1.04)
Zamora-Ros et al. [54]	Center stratified subcohort from Cohort study (EPIC-InterAct sub-cohort)	12,403 subjects W = 11,067 M = 5768 Age = 52.4 ± 9.1 year	8 European countries	24-h DR	Phenol Explorer USDA database ^(1–3)^	**Flavanols** Mean = 334 ± 286 Median = 246 5th–95th percentile = 60.9–938 **Flavonols** Mean = 24.8 ± 16.0 Median = 20.4 5th–95th percentile = 7.8–57.4 Proanthocyanidins Mean = 183 ± 140 Median = 15 15th–95th percentile = 41.7–423	↓8% type 2 diabetes risk associated with higher consumption of myricetin (Q5 = >5.38 vs. Q1 = <0.37; cut off for each quintile) (P-trend = 0.001; HR = 0.92; 95% CI: 0.88, 0.96). ↓14% type 2 diabetes risk associated with higher consumption of proanthocyanidin dimers (Q5 = >49.5 vs. Q1 = <14.1; cut off for each quintile) (P-trend = <0.003; HR = 0.94; 95% CI: 0.90, 0.99). ↓7% type 2 diabetes risk associated with higher consumption of (-)-Epicatechin (Q5 = >28.75 vs. Q1 = <6.76; cut off for each quintile) (P-trend = <0.040; HR = 0.93; 95% CI: 0.89, 0.98). ↓6% type 2 diabetes risk associated with higher consumption of (+)-Catechin (Q5 = >20.08 vs. Q1 = <5.50; cut off for each quintile) (P-trend = <0.005; HR = 0.94; 95% CI: 0.91, 0.98). ↓2% type 2 diabetes risk associated with higher consumption of (+)-Gallocatechin (Q5 = >3.45 vs. Q1 = <0.04; cut off for each quintile) (P-trend = <0.027; HR = 0.98; 95% CI: 0.97, 0.99).
Zamora-Ros et al. [55]	Cohort study (EPIC cohort)	15,258 subjects W = 9484 M = 5774 Age = 52.4 ± 9.1 year	Denmark, France, Germany, Greece, Italy, Netherlands, Norway, Spain, Sweden, and the United Kingdom	FFQs (98–266-item) Diet histories Food record	EPIC Nutrient Database based on: Phenol Explorer USDA database ^(1)^	**Total flavonoids** Mean intake = 414.9 ± 311.7 median intake = 326.7 5th percentile = 93.2 95th percentile = 1050.4 Median intake Q1 = 126.8 Q2 = 223.7 Q3 = 326.7 Q4 = 478.4 Q5 = 817.5	↓10% type 2 diabetes risk associated with higher consumption of total flavonoids (HR 0.90 [95% CI 0.72–1.07; P value trend = 0.040) ↓18% type 2 diabetes risk associated with higher consumption of flavanols (HR 0.82 [95% CI 0.68–0.99; P value trend = 0.012) ↓27% type 2 diabetes risk associated with higher consumption of flavan-3-ol monomers (HR 0.73 [95% CI 0.57–0.93; P value trend = 0.029) ↓19% type 2 diabetes risk associated with higher consumption of flavonols (HR 0.81 [95% CI 0.69–0.95; P value trend = 0.020) Conversely lignans did not show any association (HR 0.88 [95% CI 0.72–1.07] P value trend = 0.119)
Jacques et al. [56]	Cohort study (Framingham Heart Study Offspring cohort)	2915 subjects W = 1341 M = 1574 Age = 54 years CL = 53.8–54.5 year	USA	FFQs	USDA database ^(1–3)^	**Total flavonoids** Median = 210 Min = 2 Max = 1963 Median intake Q1 = 85 Q2 = 165 Q3 = 272 Q4 = 537	Total flavonoids’ intake was not significantly associated with the risk of diabetes incidence (HR = 0.89; 95% CI: 0.75–1.05) ↓ risk of diabetes incidence associated with flavanols (HR = 0.68; 95% CI: 0.54–0.86) P-trend = 0.001
Tresserra-Rimbau et al. [57]	Cohort study (PREDIMED cohort)	7172 subjects W = 3923 M = 3249 Age = 67 ± 6 year	Spain	FFQs	Phenol Explorer	**Total polyphenols** Median intake Q1 = 562 Q2 = 701 Q3 = 800 Q4 = 917 Q5 = 1170	↓ 46% CV events risk associated with higher total polyphenol intake (Q5 vs. Q1; HR = 0.54; 95%CI: 0.33–0.91) ↓ CV events risk associated with several polyphenols’ subclasses:Lignans (HR = 0.51; 95% CI: 0.30–0.86) Flavanols (HR = 0.40; 95% CI: 0.23–0.72) Hydroxybenzoic acids (HR = 0.47; 95% CI: 0.26–0.86)
Jennings et al. [58]	Cross-sectional-study	1997 subjects W = 1997 M = 0 Age = 18–76 year	UK	FFQs (131-item)	USDA database ^(1–3)^	**Total flavonoids** Mean intake = 1170 ± 639 IQR = 617–1700	Total flavonoids were not significant associated with cardiovascular outcomesTotal flavonoids inversely associated with biomarkers of insulin resistance and inflammation: ↓ HOMA-IR, insulin, hs-CRP associated with anthocyanins intake (Q5 vs. Q1) ↓ HOMA-IR, insulin, adiponectin associated with flavones intake (Q5 vs. Q1)
Ponzo et al. [59]	Cohort study	1658 subjects W = 878 M = 780 Age = 45–64 year	Italy	FFQs	USDA Database ^(1-2-3)^ extended with information from a European database	**Total flavonoids** Median intake T1 = 89 T2 = 251.4 T3 = 532.3	↓ 54% non-fatal CV events risk associated with higher flavonoid intake (T3 vs. T1; HR = 0.46; 95% CI: 0.28–0.75) ↓ non-fatal CV events risk associated with several flavonoids’ subclasses: Proanthocyanids (HR = 0.43; 95% CI: 0.27–0.70) Flavan-3-ols (HR = 0.42; 95% CI: 0.26–0.68) Anthocyanidins (HR = 0.56; 95% CI: 0.36–0.89) Flavanones (HR = 0.48; 95% CI: 0.29–0.77) Flavonols (HR = 0.53; 95% CI: 0.34–0.83) Total and subclasses of flavonoids were not significantly associated with the risk of CV mortality ↓ all-cause mortality associated with the T3 of several flavonoid subclasses: Flavan-3-ols (HR = 0.68; 95% CI 0.48–0.96) Anthocyanidins (HR = 0.66; 95% CI 0.46–0.95) Flavanones (HR = 0.59; 95% CI 0.40–0.85)
Jacques et al. [60]	Cohort study (Framingham Heart Study Offspring cohort)	2880 subjects W = 1302 M = 1578 Age = 54 year CL = 53.8–54.5	USA	FFQs	USDA database ^(1–3)^	**Total flavonoids** **Exam 5 (1991–1995)** Median = 212 25th = 124 75th = 372 **Exam 8 (2005–2008)** Median = 259 25th = 157 75th = 436	Total flavonoids’ intake was not significantly associated with the risk of incidence of CVD events (RR = 0.93; 95% CI: 0.82–1.06)
Yeon et al. [61]	Cohort study	4186 subjects W = 2575 M = 1611 Age = 40–59 year	Korea	24-h DR	USDA Database ^(1)^	**Flavanones** W = 29.24 ± 4.17 M = 21.26 ± 4.37 **Flavones** W = 0.48 ± 0.04 M = 0.36 ± 0.02 **Flavonols** W = 17.06 ± 0.55 M = 15.72 ± 0.59	↓ insulin (β-coefficient = −0.0067; p for trend = 0.0092) and HOMA (β-coefficient = −0.0016; p for trend = 0.0239) associated with flavonols intake in men ↓ insulin (β-coefficient = −0.0008; p for trend = 0.0063) and HOMA (β-coefficient = −0.0002; p for trend = 0.0119) associated with flavanones intake in women
Oh et al. [62]	Cohort study	7963 subjects W = 7963 M = 0 Age = >30 years	Korea	24-h DR	Flavonoid Korean Database	**Total flavonoids** Mean Intake: Normal fasting glucose group = 107.40 ± 1.69 Type 2 diabetes mellitus group = 97.81 ± 8.11	↓prevalence of type 2 diabetes associated with intake of flavones above the 25th percentile (≥0.25 mg/day) compared with intake below the 25th percentile (OR = 0.593, 95% CI: 0.414–0.847)
Goetz et al. [63]	cohort study	20,024 subjects W = 11,253 M = 8771 Age = >45 years	US	FFQs (107-item)	USDA database ^(1–3)^	**Total flavonoids** Median intake (range) Q1 = 34.3 (<50.8) Q2 = 66.6 (50.9–83.4) Q3 = 102.9 (83.5–127.0) Q4 = 156.9 (127.1–208.3) Q5 = 296.8 (≥ 208.4)	↓risk of incident acute ischemic stroke (HR = 0.72; 95% CI: 0.55, 0.95; P-trend = 0.03) was associated with flavanone intake, but not total or other flavonoid subclasses. Associations did not differ by sex race, or region for any flavonoid measure.
Goetz et al. [64]	Cohort study	16,678 subjects W = 9798 M = 6880 Age = >45 years	US	FFQs (107-item)	USDA database ^(1–3)^	**Total flavonoids** W: Mean intake = 234 Median intake = 131 M: Mean intake = 227 Median intake = 131	↓incident CHD associated with consumption of anthocyanidin and proanthocyanidin. Anthocyanidins Q1 vs. Q5; HR = 0.71; 95% CI: 0.52–0.98; P-trend = 0.04; proanthocyanidins Q1 vs. Q5; HR = 0.63; 95% CI: 0.47–0.84; P-trend = 0.02). There was no significant effect modification by age, sex, race, or region of residence
Miranda et al. [65]	Cohort study	550 subjects W = 346 M = 204 Age = 20–59 years Age older adults = >60 years	Brazil	2 24-h DR	Phenol Explorer	**Total polyphenols** Mean intake = 392.6 Median intake = 360.6	↓hypertension associated with highest tertiles of some classes of polyphenols: tyrosols (OR = 0.33; 95% CI 0.18–0.64), alkylphenols (OR = 0.45; 95% CI 0.23–0.87), lignans (OR = 0.49; 95% CI 0.25–0.98), as well as stilbenes (OR = 0.60; 95% CI 0.36–0.98), and other polyphenols (OR = 0.33; 95% CI 0.14–0.74). ↓hypertension associated with middle tertiles of total polyphenols and phenolic acids.There was no significant association for total flavonoids
Cassidy et al. [66]	Cohort study (HPFS cohort)	43,880 subjects M = 43,880 W = 0 Age = 32–81 years	UK	FFQs	USDA database ^(1)^	**Anthocyanins** Q1 = 1.9 Q2 = 4.5 Q3 = 7.8 Q4 = 13.7 Q5 = 26.3 intake range = 0–613 IQR = 3.9–15.7 **Flavanones** Q1 = 7.5 Q2 = 23.6 Q3 = 43.5 Q4 = 64.5 Q5 = 103.9 intake range = 0–728 IQR = 18.8–70.9	↓total or fatal MI risk associated with higher anthocyanin intake (HR = 0.87; 95% CI: 0.75–1.00; P = 0.04; P-trend = 0.098); this association was stronger in normotensive participants (HR = 0.81; 95% CI: 0.69–0.96; P-interaction = 0.03). Anthocyanin intake was not associated with stroke risk. ↓ischemic stroke associated with higher flavanone intake (HR = 0.78; 95% CI: 0.62–0.97; P = 0.03, P-trend = 0.059); with the greatest magnitude in participants aged > 65 years (P-interaction = 0.04). Flavanone intake was not associated with MI or total stroke risk
Kim et al. [67]	Cohort study	4042 subjects W = 1970 M = 2072 Age = >19 years	US	2 24-h DR	USDA Database ^(1-2-3)^	**Total flavonoids** Mean intake Q1 = 12.5 Q2 = 59 Q3 = 197.6 Q4 = 585.5	Changes in percentages of cardiovascular risk factors with a 100% increase in flavonoid intake: ↑ 0.54% HDL-cholesterol associated with higher total flavonoid intake ↓ 1.25% TAG and ↓ 1.60% TAG:HDL-cholesterol ratio associated with anthocyanidin intake ↓ 1.31% TAG and ↓ 1.83% TAG:HDL-cholesterol ratio associated with total flavonoid intake ↓ 3.18% insulin and ↓ 3.10% HOMA-IR were associated with flavone intake ↓ 3.11% insulin and ↓ 4.01% HOMA-IR were associated with isoflavone intake ↓ 0.60% BMI associated with anthocyanidin intake
Rizzi et al. [68]	Cohort study	443 subjects W = 175 M = 268 Age = 20–85 years	Italy	24-h DR	USDA Database ^(1)^ Phenol Explorer EIO Database	**Total polyphenols** range intake T1 = 99.4–804.5 T3 = 1288.0–4342.2	High polyphenols intake was not associated with significant differences in the lipid profile compared with low polyphenols intake
Grosso et al. [69]	Cohort study (HAPIEE study)	5806 subjects W = 3075 M = 2731 Age = 45–69 years	Poland	FFQs (148-item)	Phenol Explorer	**Total polyphenols** Mean intake Q1 = 1026.7 ± 212 Q2 = 1469.6 ± 102.2 Q3 = 1872.6 ± 136.7 Q4 = 2632.1 ± 608	↓ 32% of risk of type 2 diabetes in the whole population associated with highest intake of total polyphenol (Q4 vs. Q1)
Witkowska et al. [70]	Cohort study	2599 subjects W = 2599 M = 0 Age = 20−74 years	Poland	24-h DR (367-item)	Phenol Explorer	**Total polyphenols** Mean intake Q1 = 948.2 ± 236 Q2 = 1523.2 ± 142 Q3 = 2016.3 ± 154 Q4 = 2975.8 ± 724	↓ 1.1% odds ratio of CVD in postmenopausal women with higher dietary polyphenol intake (per 100 mg/day)
Grosso et al. [71]	Cohort study (HAPIEE study)	8821 subjects W = 4530 M = 4291 Age = 50–65 years	Poland	FFQs (148-item)	Phenol Explorer	**Total polyphenols** n.a.	↓ metabolic syndrome associated with the highest quartile of polyphenol intake (OR = 0.80; 95% CI: 0.64–0.98 and OR = 0.70; 95% CI: 0.56–0.86 for both men and women, respectively). ↓ blood pressure, waist circumference, high lipoprotein cholesterol, and triglycerides associated with high total polyphenol intake in women. ↓ fasting plasma glucose associated with high total polyphenol intake in both genders.
Sohrab et al. [72]	Cohort study	1265 Subjects W = 711 M = 554 Age = 19–74 years	Iran	FFQs	Phenol Explorer	**Total polyphenols** Median Intake (range) T1 = 827 (≤1128) T2 = 1425 (1129–1819) T3 = 2459 (≥1820) **Total flavonoids** Median intake (range) T1 = 38.4 (≤52.8) T2 = 69.5 (52.9–88.4) T3 = 115.1 (≥88.5)	Total polyphenols were not significant associated with metabolic syndrome ↓ 31% metabolic syndrome risk (OR = 0.69; 95% CI: 0.48–0.98, P-trend: 0.04) associated with total flavonoid intake (T3 vs. T1)
Mendonça et al. [73]	Cohort study (SUN cohort)	17,065 Subjects W = 10,358 M = 6707 Age = 20–89 years	Spain	FFQs 136-item	Phenol Explorer USDA database	**Total polyphenols** Mean Intake Q1 = 396 (±134) Q2 = 526 (±149) Q3 = 653 (±149) Q4 = 812 (±156) Q5 = 1248 (±405) **Total flavonoids** Mean Intake Q1 = 186 (±72) Q2 = 234 (±86) Q3 = 302 (±97) Q4 = 424 (±105) Q5 = 772 (±330)	Total polyphenols were not significant associated with cardiovascular events (HR = 0.61; 95% CI: 0.33–1.13 P for trend 0.28) Total flavonoids were not significant associated with cardiovascular events (HR = 0.53; 95% CI: 0.29–0.98 P for trend 0.09)

Legend: n.a. = not available; 24-h DR = 24 h dietary recall; M = men; W = women; FR = food record; FFQ = food frequency questionnaire. ^(1)^ = USDA database (Flavonoids) USDA Database for the Flavonoid Content of Selected Foods, Release 2.1. Internet. 2007 Ref Type: Electronic Citation. ^(2)^ = USDA database (isoflavones) U. S. Department of Agriculture. Beltsville: MD: USDA; 2008. Database for the Isoflavone Content of Selected foods. Ref Type: Electronic Citation. ^(3)^ = USDA database (proanthocyanidins) USDA Database for the Proanthocyanidin Content of Selected Foods. Internet. 2004 Ref Type: Electronic Citation. a = Milder et al. Lignan contents of Dutch plant foods: a database including lariciresinol, pinoresinol, secoisolariciresinol and matair- esinol. Br J Nutr 2005.; Valsta et al. Phyto-estrogen database of foods and average intake in Finland. Br J Nutr 2003.; Mazur et al.Adlercreutz H. Lignan and isoflavonoid concentrations in tea and coffee. Br J Nutr 1998.; Mazur et al. Natural and anthropogenic environ- mental oestrogens: the scientific basis for the risk assessment. Naturally occurring oestrogen in food. Pure Appl Chem 1998.

**Table 3:** Association between polyphenol intake and all-cause/cardiovascular mortality.

References	Type of Study	Population Characteristics	Country	Dietary Assessment - n° Food-Containing Items	Polyphenol Database Source n° Food Items	Estimated Polyphenol Intake (mg/day) mean± sd/quantile/ min-max/IQR	Overall Results/Association with Outcome
McCullough et al. [74]	Cohort study (American Cancer Society’s CPS-II Nutrition Cohort Study)	98,469 subjects W = 60,289 M = 38,180 Mean Age W = 70 years Mean Age M = 69 years	USA	FFQs (152 food items)	USDA database ^(1-2-3)^ other research publications	**Total flavonoids** Mean intake (energy-adjusted) **Men** Mean intake = 268 Median intake = 203 10th–90th percentile = 99–498 **Women** Mean intake = 268 Median intake = 201 10th–90th percentile = 92–522	**Cardiovascular mortality** **Age-adjusted model:** Inverse association were observed for high total flavonoid, anthocyanidins (median 22.2 (≥16.7) mg/day), flavan-3-ols (median 63.7 (≥37.2) mg/day), flavones (median 3.0 (≥2.1) mg/day), flavonols (median 27.2 (≥20.6) mg/day), proanthocyanidins (median 379.4 (≥253.6) mg/day) and isoflavones (median 0.713 (≥0.142) mg/day) in both the sex. Inverse association for flavanones (median 49.9 (≥35.4) mg/day) in women. **Multivariable-adjusted model**^3^: No association in men. Inverse association for high total flavonoid, anthocyanidin, flavan-3-ol intake in women. Subjects with high total flavonoid consumption (median 512.5 (≥359.7) mg/day) showed a low risk of death (−18%) in both the sex.Inverse association for high anthocyanidin, flavan-3-ol, flavones, flavanol and proanthocyanidin intake by considering women + men. **Ischemic heart disease mortality** **Age-adjusted model**: Inverse association for high anthocyanidin and flavone intake in both the sex. Inverse association for high total flavonoid intake in men and women + men; high flavanone intake in women + men; high flavanol intake in women and women + men; high proanthocyanidin intake in women + men; high isoflavone intake in men and women + men. **Multivariable-adjusted model**^3^: Inverse association for high flavone intake in women and women + men **Stroke mortality** **Age-adjusted model**: Inverse association for high total flavonoid intake in men, and high flavones intake in men and women + men. **Multivariable-adjusted model**^3^: Inverse association for high total flavonoid intake in men
Zamora-Ros et al. [75]	Cohort study(Invecchiare in Chianti study)	807 subjects W = 447 M = 360 Age = 74.3 ± 6.9 years Survived W = 313 M = 240 Age = 71.8 ± 5.3 years Died W = 134 M = 140 Age = 79.2 ± 7.2 years	Italy	FFQs (Italian version) Urinary polyphenol assessment	Phenol Explorer USDA database	**Total polyphenols** Mean intake = 594 ± 196 **Survived** Mean intake = 600 ± 201 **Died** Mean intake = 584 ± 185	No association between total dietary polyphenols and all-cause mortality
Zamora-Ros et al. [76]	Cohort study (EPIC Spain Study)	40,622 subjects W = 25,298 M = 15,324 Age = 29–69 years	Spain	FFQs computerized diet history questionnaire developed and validated specifically for the EPIC study in Spain	USDA database Phenol-explorer UK Food Standards Agency (10% missing values in 1877 food items)	**Total flavonoids** Mean intake = 387.3 ± 280.2 Median intake = 329.8 25th percentile = 218.4 75th percentile = 489.6 **Total lignans** Mean intake = 1.0 ± 0.5 Median intake = 0.9 25th percentile = 0.7 75th percentile = 1.2	**Multivariable-adjusted model**^2^: subjects with high flavanones (>51.3 mg/day), flavanols (>28.0 mg/day) and total flavonoids intake (>447.8 mg/day) showed a low risk of all-cause mortality (flavanones: 0.60 (95% CI = 0.38–0.94) and flavonols: 0.59 (95% CI = 0.40–0.88). This reduction was due entirely to a decrease in mortality from CVD. Proanthocyanidins were the most important contributor (66%) to total flavonoid intake, followed by flavanones (11%), flavan-3-ol monomers (9%), anthocyanidins (7%), and flavonols (6%), flavones (1%), isoflavones (0.1%), and theaflavins (<0.1%). No evidence of an association between dietary flavonoid or lignan intake and mortality from cancer or other causes.
Ivey et al. [77]	Cohort study	1063 Subjects W = 1063 Age > 75 years	Australia	FFQs developed by the AntiCancer Council of Victoria	USDA	**Total flavonoids** **Flavonol**: 31 ± 14 **Flavan-3-ol**: 431 ± 279 **Proanthocyanidin**: 215 ± 147 Flavone: 3 ± 2 **Flavanone**: 53 ± 38 **Anthocyanidin**: 37 ± 26 **Isoflavone**: 5 ± 6	**Unadjusted model**: Subjects with high intake of total flflavonol (>35 mg/day), flflavan3-ol (>563 mg/day), flavone (>3 mg/day) and flavanone (>61 mg/day) showed a reduced risk of atherosclerotic vascular disease mortality. **Age- and energy-adjusted model and multivariate-adjusted model**^4^: Subjects with high intake of total flflavonol (>35 mg/day), flflavan3-ol (>563 mg/day) showed a reduced risk of atherosclerotic vascular disease mortality No association was observed for the other flavonoid subclasses Tea contributed 59% of total flavonoid intake; the major contributors were flavonols (65%) and flavan-3-ols (93%). **Multivariate-adjusted model**^4^: Subjects with high intake of flavonols derived from tea and non-tea sources (≥12 mg/day and ≥27 mg/d, respectively) showed a low risk of atherosclerotic vascular disease mortality.
Ivey et al. [78]	Cohort study	1063 subjects W = 1063 M = 0 Age > 75 years	Australia	FFQs (93 items) Beverage questionnaire (to assess tea and coffee consumption)	Phenol Explorer (47 foods recorded as not containing flavonoids or not present in the database) USDA database ^(1-2-3)^ (19 foods recorded as not containing flavonoids or not present in the database)	**Total flavonoids (PE)** Mean intake = 674 ± 326 Median intake = 648 IQR = 449–872 **Total flavonoids (USDA)** Mean intake = 696 ± 322 Median intake = 668 IQR = 468–889	Subjects with high total flavonoid intake (≥813 mg/day USDA; ≥788 mg/day PE) showed a low risk of all-cause mortality and cardiovascular mortality
Ponzo et al. [59]	Cohort study	1658 subjects W = 878 M = 780 Age = 45–64 year	Italy	FFQs	USDA Database ^(1-2-3)^ extended with information from an European database	**Total flavonoids** Median intake T1 = 89 T2 = 251.4 T3 = 532.3	Total and subclasses of flavonoids were not significantlyassociated with the risk of CV mortality The third tertile of flavan-3-ols (HR = 0.68; 95% CI 0.48–0.96), anthocyanidins (HR = 0.66; 95% CI 0.46–0.95) and flavanones (HR = 0.59; 95% CI 0.40–0.85) was inversely associated with all-cause mortality
Dower et al. [79]	Cohort study (Zutphen Elderly Study)	774 subjects W = 0 M = 774 Age = 65–84 years	The Netherlands	Cross-check dietary history (adapted for the Dutch setting) ^5^	Monomeric flavan-3-ol contents of 120 commonly consumed plant foods and beverages were determined with the use of reverse-phase HPLC with ultraviolet and fluorescence detection. (−)-epicatechin, (+)-catechin, (−)-epigallocatechin,(−)-epicatechin gallate (ECg), (−)-epigallocatechin gallate (EGCg), and (+)-gallocatechin concentrations were determined as reported in previous published papers ^6^	**Total epicatechins** Mean intake = 15.2 ± 7.7 Range intake = 0.01–60.6	**Coronary heart disease mortality** Subjects with high epicatechin intake (>18 mg/day) showed a low (−38%) risk of CHD mortality **Cardiovascular disease mortality** Subjects with high epicatechin intake (>18 mg/day) showed a low (−46%) risk of CVD mortality in men with prevalent CVD but not in men who were free of CVD The major dietary sources of epicatechin intake were tea (7.8 mg/day; 51% of total epicatechin intake), apples (4.3 mg/day; 28% of total epicatechin intake), cocoa (1.1 mg/day; 7% of total epicatechin intake), and other sources (2.0 mg/day; 13% of total epicatechin intake)
Ivey et al. [80]	Cohort study (Nurses’ Health Study II)	93,145 subjects W = 93.145 M = 0 Age = 25–42 years	USA	FFQs	USDA database	**Total flavonoids** Mean intake = 379 ± 374	**Age-adjusted model**: subjects with high total flavonoids intake (≥518 mg/day) showed a 19% reduction of overall mortality in the 18-year follow-up period. Subjects with high flavan-3-ols (≥86 mg/day), proanthocyanidins (≥356 mg/day) and anthocyanin intake (≥17 mg/day) showed a low risk of mortality for CVD and other causes. **Multivariable adjusted model**^1^: no association High consumption (more than once per week) of red wine, tea, peppers, blueberries and strawberries was associated with reduced risk of total and cause-specific mortality.
Zhang et al. [81]	Cohort study	6235 subjects with breast cancer W = 6235 M = 0 Age = 51.8 ± 10.6 years	USA, Canada, Australia	FFQs	USDA database	**Total isoflavones** Mean intake: 1.8 ± 3.9 Median intake: 0.7 IQR intake: 1.2	Quartile 4 (≥1494 mg/day) associated with a 21% decrease in all-cause mortality. This result was limited to women with negative tumor hormone receptors and those not treated with hormonal therapy for breast cancer
Pounis et al. [82]	Cohort study (Moli-sani cohort Study)	21,302 subjects W = 10,980 M = 10,322 Age = 54–56 years	Italy	FFQs	Eurofir-eBASIS FCTs USDA database ^(1-2-3)^	Data reported as polyphenol antioxidant content (PAC)-score ^6^ (−28 to 28)	**Risk for all-cause mortality** Women: low risk for high intake of flavones (>1.12 mg/day), flavanones (>46.5 mg/day), isoflavones (>32.7 mg/day), and lignans (>116.1 mg/day) had a low risk. **After adjustments for potential confounders (model 4)**^7^: the effects remained significant for Q4 (4–13) and Q5 (>13) of PAC-score Men: low risk for some quintile of intake; flavonols (Q2: 11.2–15.1 mg/day and Q5: >25.8 mg/day), flavones (Q3: 0.61–0.81 mg/day), flavanones (Q2: 22–29 mg/day), isoflavones (Q2: 16.5–21 mg/day, Q3: 21–25.7 mg/day and Q5: >32.7 mg/day), lignans (Q3: 72.8–90.3 mg/day). **After adjustments for potential confounders (model 4)**^8^: the effects remained significant for Q2 (−13 to −4), Q3 (−4 to 4) and Q4 (4–13) of PAC-score **Vascular causes** Women: no association Men: low risk of mortality for Q2 (−13 to −4) and Q3 (−4 to 4) **Other causes** Women and men at Q2 (−13 to −4) and Q4 (4–13) of PAC-score showed a low mortality risk from other causes

Legend: n.a. = not available; 24-h DR = 24 h dietary recall; M = men; W = women; FR = food record; FFQ = food frequency questionnaire. ^1^ BMI, smoking status, menopausal status, family history of diabetes/cancer/myocardial infarction, multivitamin supplement use, aspirin use, race, type 2 diabetes, hypercholesterolaemia, hypertension, physical activity, alcohol consumption and energy intake and the Alternative Health Eating Index (minus alcohol) score. ^2^ Cox proportional hazards regression models were stratified by center, age (1 year) and sex and adjusted for BMI, education level, physical activity, tobacco smoking, alcohol lifetime, total energy, vitamin C and fiber intake. ^3^ Age, smoking, beer and liquor intake, history of hypertension, history of cholesterol, family history of myocardial infarction, BMI, physical activity, energy intake, aspirin use, hormone replacement therapy (in women only), and sex (in combined model only) by using Cox proportional hazards regression. ^4^ age, previous CVD, previous diabetes, energy expended in physical activity and history of smoking. ^5^ Keys A et al., Acta Med Scand Suppl 1966;460:1–392. ^6^ Arts ICW et al., J Agric Food Chem 2000;48:1752–7; Arts ICW et al., J Agric Food Chem 2000;48:1746–51. ^7^ Pounis et al., European Journal of Clinical Nutrition 2016; 70;338–345. ^8^ Age, energy intake, smoking habits, social status, physical activity level and INFLA-score. Eurofir-eBASIS: European Food Information Resource—Bioactive Substances in Food Information Systems; FCTs: Italian Food Composition Tables; ^(1)^ USDA database (Flavonoids) USDA Database for the Flavonoid Content of Selected Foods, Release 2.1. Internet. 2007 Ref Type: Electronic Citation. ^(2)^ USDA database (isoflavones) U. S. Department of Agriculture. Beltsville: MD: USDA; 2008. Database for the Isoflavone Content of Selected foods. Ref Type: Electronic Citation. ^(3)^ USDA database (proanthocyanidins) USDA Database for the Proanthocyanidin Content of Selected Foods. Internet. 2004 Ref Type: Electronic Citation.

**Table 4:** Polyphenol intake and other outcomes.

References	Type of Study	Population Characteristics	Country	Dietary Assessment - n° Food-Containing Items	Polyphenol Database Source n° Food Items	Estimated Polyphenol Intake (mg/day) mean ± SD/quantile/min-max/IQR	Overall Results/Association with Outcome
Fisher et al. [83]	Analytical	19 subjects W = 11 M = 8 Age = 72 ± 7 years	US	FFQs	USDA database ^(1–3)^ (22 food item)	**Total flavonoids** Median intake = 2428 mg/week Median = 347 Q1–Q4 = 1242–4789 mg/week	Habitual dietary intake of flavonoids was associated with higher endothelial function evaluated as reactive hyperemia (RH)-PAT response. Subjects with habitual flavonoid intake (>4500 mg/week) had significantly higher (RH)-PAT response
Ivey et al. [84]	Prospective	948 subjects W = 948 M = 0 Age = ≥75 years	Australia	FFQs	USDA database ^(1-2-3)^	**Total Proanthocyanidins** Mean intake = 215 ± 147 Min-max = 18–1728	Over 50% of total proanthocyanidin intake were from fruit (89 ± 63 mg/day), chocolate (43 ± 75 mg/day), and alcoholic beverages (32 ± 86 mg/day). Subjects with habitual proanthocyanidin intake (≥229 mg/day) had lower risk of moderate chronic kidney insufficiency and renal failure events
Zhang et al. [85]	Cross-sectional	3317 subjects W = 2239 M = 1078 Age = 60.2 years	China	FFQs (79-item)	USDA database ^(1–3)^ Hong Kong database of isoflavones ^1^	**Total flavonoids** Median intake **W(Q1)** = 53.3 IQR = 40.5–66.3 W(Q2) = 110.0 IQR = 92.2–132.1 W(Q3) = 232.4 IQR = 194.8–274.4 **W(Q4)** = 486.9 IQR = 402.2–584.4 **M(Q1)** = 63.1 IQR = 44.9–94.6 M(Q2) = 207.9 IQR = 174.4–237.2 M(Q3) = 351.8 IQR = 297.6–392.2 **M(Q4)** = 555.3 IQR = 479.6–618.2	High total flavonoid intake (Q4 vs. Q1) was associated with higher bone mineral density (BMD) in women, but not in men. A dose dependent positive relationship was found for all BMD measured sites. In addition, a significant association was found also for flavonoid subclasses (flavonols, flavan-3-ols, flavones, and proanthocyanidins)
Urpi- Sarda et al. [86]	Cross-sectional (Invecchiare CHIANTI Study)	811 subjects W = 446 M = 364 Age = >65 years	Italy	FFQs	Phenol explorer USDA database	**Total polyphenols** Mean intakeAll (N = 811) = 595.2 ± 195.6 Non-frail (n = 418) = 608.5 ± 199.8 Prefrail (n = 321) = 587.3 ± 195.9 Frail (n = 72) = 550.5 ± 158.7 1T < 509.2 2T = 509.2–645.2 3T < 645.2	No association between total dietary polyphenols and frailty and pre-frailty in older subjects
Rabassa et al. [95]	Cross-sectional (Invecchiare CHIANTI Study)	652 subjects W = 361 M = 291 Mean Age = 73	Italy	FFQs	Phenol explorer USDA database	**Total polyphenols** Median intake All (n = 652) = 574 IQR = 472–701 1T = 430 IQR = 354–470 2T = 574 IQR = 543–610 3T = 766 IQR = 701–855	No association between total dietary polyphenols and any cognitive test in older subjects
Myers et al. [87]	Prospective	1188 subjects W = 1188 Age = >70 years	Australia	FFQs Beverage questionnaire	USDA database ^(1-2-3)^	**Total flavonoids** Median intake **Tea Low consumer** = 266 IQR = 191–361 **Tea Moderate consumer** = 845 IQR = 672–959 **Tea High consumer** = 1570 IQR = 1325–1915	Higher intake of black tea and flavonoids was associated with lower hospitalization (30–40% reduction) for fractures in older women at high risk
Ma et al. [88]	Case-control	249 subjects (cases) 66 subjects (controls) W = 182 M = 133 Age = 50–70 years	China	FFQs 3 24-h DR	USDA database	**Total flavonoids** **Cases** Median intake = 51.13 IQR = 38.06–64.21 **Controls** Median intake = 64.92 IQR = 53.66–75.61	Total dietary anthocyanidin, flavan-3-ol, flavanone, flavone, and flavonol intake was not associated with age related cataract risk. Only quercetin and isorhamnetin intake appeared to be associated with the risk in this population
Rabassa et al. [89]	Cross-sectional (Invecchiare CHIANTI Study)	368 subjects W = 199 M = 169 Age = >65 years	Italy	FFQs	USDA database ^(1–3)^ Phenol explorer 236 food items	**Total polyphenols** **Baseline** Median intake = 556 IQR = 462–682 **3-year follow-up** Median = 539 IQR = 429–656 **6-years** Median = 513 IQR = 415–619 **9-years** Median = 500 IQR = 407–595	Total dietary polyphenol (TDP) intake was higher in older subjects and women with higher physical activity level. No association between TDP and physical performance decline was found
Garcia-Larsen et al. [90]	Cross-sectional (GA^2^LEN study)	2599 subjects W = 1516 M = 1083 Age = 47.2 ± 14.5 years	Denmark Finland Sweden UK Portugal Belgium Germany Netherlands Poland	FFQs (250-item)	USDA database ^(1–3)^	**Total flavonoids** Median intake = 291.2 IQR = 126.8–569.4	Total flavonoid intake and pro-anthocyanidins was positively associated with a good ventilatory function (forced vital capacity), while a negative association with spirometric restriction was found in the cohort. In particular, subjects with total flavonoid intake at the highest quintile had a 42% lower risk of reduced forced vital capacity
Gopinath et al. [91]	Cohort study (Blue Mountains Eye Study)	2856 subjects W = 1597 M = 1259 Age = ≥ 49 years	Australia	FFQs (145-item)	USDA database ^(1-2-3)^	**Total flavonoids** Median intake = 875 Q1 ≤ 410.6 Q2 = 412.4–881.5 Q3 = 881.6–1232.3 Q4 ≥ 1232.4	Total flavonoids and subclasses (e.g., flavonols and flavanones), were associated with age-related macular degeneration (AMD) among older adults. The consumption of oranges and orange juice, contributing to total flavanone intake, was found to significantly affect AMD risk
Pounis et al. [92]	Cross-sectional (Moli-sani study)	9659 subjects W = 4551 M = 5108 Age = ≥35 years	Italy	FFQs (164-item)	Eurofir–e BASIS USDA database ^(1-2-3)^	**Flavonols** Median intake (Q1–Q3) W = 15.4 (11.1–21.2) M = 19.1 (14.1–26.0) **Isoflavones** Median intake (Q1–Q3) W = 23.3 (17.9–31.0) M = 23.7 (18.1–31.1) **Flavones** Median intake (Q1–Q3) W = 0.77 (0.53–1.10) M = 0.65 (0.44–0.95) **Flavanones** Median intake (Q1–Q3) W = 31.1 (22.9–42.1) M = 35.0 (26.1–45.9) **Flavanols** Median intake (Q1–Q3) W = 41.6 (24.4–73.0) M = 66.1 (36.3–108.8) **Anthocyanidins** Median intake (Q1–Q3) W = 145.3 (99.8–209.3) M = 148.0 (101.9–216.3) **Lignans** Median intake (Q1–Q3) W = 82.7 (61.1–109.8) M = 81.2 (61.1–107.2)	Higher polyphenol intake was associated with better pulmonary function (forced vital capacity, and forced expiratory volume in the first second) in the population under study. A potential anti-inflammatory activity of polyphenols was hypothesized in men where a reduction in C-reactive protein and white blood cells was observed
Lefevre-Arbogast et al. [93]	Cohort study (The 3C Bordeaux cohort)	1329 subjects W = 824 M = 505 Mean Age = 75.8 years	France	24-h DR	Phenol Explorer	**Total polyphenols** Mean intake **All subjects** = 1071 ± 570 **Incident dementia** = 1029 ± 542 (n = 256) **No dementia** = 1081 ± 576 (n = 1073)	Polyphenol intake was associated with a decreased risk of all-cause dementia and of Alzheimer disease (AD) over 12 years. Subjects in the higher quintile of intake had a ≈ 50% lower risk of both dementia and AD. The pattern of polyphenol intake associated with the reduced risk was characterized by flavonoids (e.g., dihydroflavonols, anthocyanins, isoflavonoids, and flavanones), stilbenes (including resveratrol), lignans, and additional isolated polyphenols (hydroxybenzaldehydes, naphthoquinones, and furanocoumarins)
Segovia-Siapco et al. [94]	Cross-sectional (The Teen Food and Development Study)	248 subjects W = 0 M = 248 Age = 12–18 years	USA	Web-FFQs (151-item)	Nutrition Data Systems for Research (NDS-R) Specific database ^2^	**Total Isoflavones** Mean intake = 22.1 Min and max = 18.3–26.0	Moderate (3–20 mg/day) and high (>20 mg/day) consumers of soy isoflavones nearly follow the same pattern for pubertal development. Whether soy isoflavones play a role in the rate of maturation and sequencing of pubertal development in boys cannot be determined based on our study findings

Legend: n.a. = not available; 24-h DR = 24 h dietary recall; M = men; W = women; FR = food record; FFQ = food frequency questionnaire; sFFQs = semi-quantitative FFQ. ^(1)^ USDA database (Flavonoids) USDA Database for the Flavonoid Content of Selected Foods, Release 2.1. Internet. 2007 Ref Type: Electronic Citation. ^(2)^ USDA database (isoflavones) U. S. Department of Agriculture. Beltsville: MD: USDA; 2008. Database for the Isoflavone Content of Selected foods. Ref Type: Electronic Citation. ^(3)^ USDA database (proanthocyanidins) USDA Database for the Proanthocyanidin Content of Selected Foods. Internet. 2004 Ref Type: Electronic Citation. ^1^ Chan SG, Murphy PA, Ho SC, Kreiger N, Darlington G, So EK, Chong PY (2009) Isoflavonoid content of Hong Kong soy foods. J Agric Food Chem 57:5386–5390. ^2^ Jaceldo-Siegl K, Fraser GE, Chan J, Franke A, Sabaté J (2008).
